# QM/MM Reveals Coordination Shift behind Early Lanthanide Preference in Methanol Dehydrogenase XoxF

**DOI:** 10.1021/acs.jpcb.6c03103

**Published:** 2026-08-27

**Authors:** Emiliano Isaías Alanís-Manzano, José Luis Velázquez-Libera, César Millan Pacheco, Alejandro Ramírez-Solís, Humberto Saint-Martin

**Affiliations:** † Instituto de Ciencias Físicas, 7180 Universidad Nacional Autónoma de México, Cuernavaca Morelos 62210, México; ‡ Departamento de Bioinformática, Centro de Bioinformática, Simulación y Modelado (CBSM), Facultad de Ingeniería, 28066 Universidad de Talca, Talca 3460000, Chile; § Facultad de Farmacia, 104061 Universidad Autónoma del Estado de Morelos, Cuernavaca, Morelos 62209, México; ∥ Centro de Investigación en Ciencias, Universidad Autónoma del Estado de Morelos, Cuernavaca, Morelos 62209, México

## Abstract

Lanthanides serve as essential catalytic cofactors in methanol dehydrogenase (MDH), yet the structural and energetic basis for the biological preference for early lanthanides (La–Gd) remains poorly understood. Here, we present a systematic Quantum-Mechanics/Molecular-Mechanics (QM/MM) study of the full Ln series (La–Lu) using the ωB97X-D4rev exchange-correlation functional. Structurally, we identify a substrate-dependent coordination switch: in the Michaelis–Menten complex, the biologically relevant light Ln (La–Gd) maintains a 10-coordinate geometry, whereas a discrete transition to a 9-coordinate state occurs from Tb onward, driven by the shift of the essential Asp301 from bidentate to monodentate coordination mode. Notably, this shift is absent in the substrate-free enzyme, indicating that substrate binding enforces the steric crowding that disfavors heavier metals. Energetic analysis reveals that within the biologically relevant window (La–Gd), substrate affinity is mostly uniform, while enzyme binding affinity exhibits a clear and steady preference for the lighter metals. These results demonstrate that biological lanthanide selection in MDH arises from a combination of higher binding affinity for early lanthanides and their ability to preserve a catalytically competent 10-coordinate geometry, rather than from differential substrate binding. Taken together, our findings provide a structural and energetic framework for understanding the coordination properties of the lanthanide series in the active site of the MDH and may guide further QM/MM studies on the reaction mechanism of this enzyme as well as the engineering of lanthanide-dependent biocatalysts.

## Graphical abstract

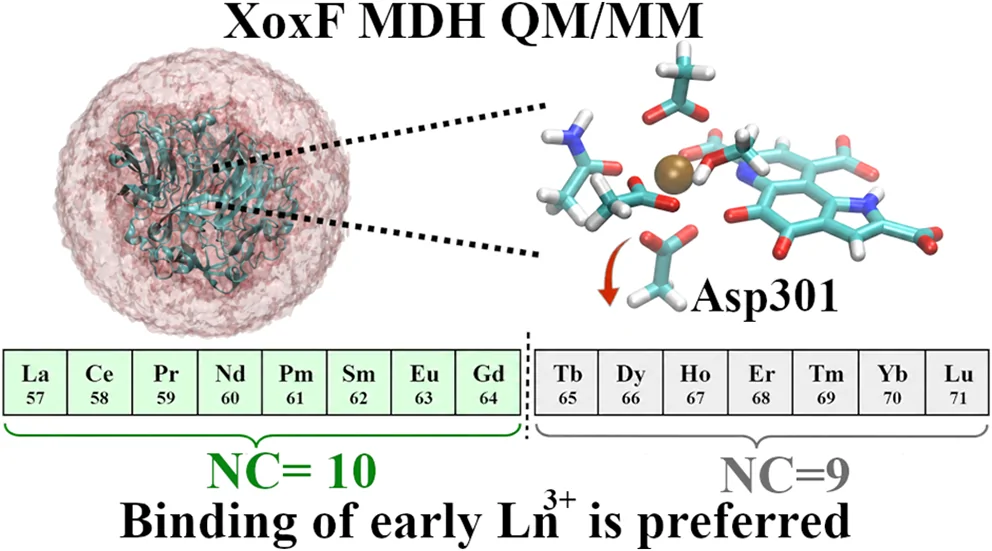

## Introduction

Lanthanides (Lns) have long been considered technologically critical elements, essential components of high-performance magnets, lasers, and electronics.^1^ However, until recently, they were considered biologically inert, due to the low solubility of their oxides and phosphates and the fact that they are not essential in the biochemistry of complex multicellular organisms.^2^ This view changed dramatically in 2011, when lanthanides were first identified as cofactors in the metabolism of methylotrophic bacteria.^1,3,4^

Lns are now recognized as biologically relevant metals, acting as cofactors of methanol dehydrogenase (MDH) in bacteria that utilize methane as their sole energy and carbon source.^5–8^ MDH catalyzes the oxidation of methanol to formaldehyde, an essential process for bacterial metabolism. To do so, Ln^3+^ ions are required as well as a pyrroloquinoline quinone (PQQ) redox cofactor at the active site. Ln^3+^ ions act as Lewis acids in MDH, a role commonly fulfilled in other organisms by Ca^2+^ ions. The active sites of Ln- and Ca-MDH differ only by the presence of one additional aspartate in the coordination sphere of Ln^3+^.^9^ Although, the lanthanide-dependent MDH (XoxF) is evolutionarily older and more widespread in nature than its Ca^2+^-dependent counterpart (MxaF), the reaction mechanism and the reason why Lns are preferred over calcium in these enzymes is not yet fully understood.

Not all Lns promote this enzymatic activity equally.^10–13^ The lighter Lns (La^3+^–Gd^3+^) sustain high MDH activity, whereas heavier Lns, despite their greater Lewis acidity, are markedly less effective. Notably, Yttrium and Scandium-which share the crustal abundance of early Lns-have been tested in kinetic assays and also fail to promote high MDH activity,^5,14^ indicating that availability alone cannot account for the observed selectivity.^5^ The mechanism by which specific lanthanides modulate Ln^3+^-MDH activity therefore remains an open question with direct implications for the biological relevance of the lanthanide series.

The effect of metal substitution on the active site has been explored through Density Functional Theory (DFT) studies using cluster models^12,15–17^ and one classical molecular dynamics study.^18^ However, these efforts have yielded contradictory trends and thus a coherent picture of how the specific type of the Ln^3+^ ion shapes the active site properties has yet to emerge. For instance, Tsushima^18^ reports a shift in the coordination mode of the PQQ cofactor, from tridentate with La^3+^ and Eu^3+^ to monodentate with Yb^3+^. On the other hand, Russo and collaborators^16^ reported a constant coordination number (CN) throughout the series. Cluster models, by construction, neglect the broader protein environment, which may play a decisive role in determining the coordination properties of the metal center. Furthermore, pure MD-based studies rely on the force field approximation which might be unreliable to treat highly charged Ln^3+^ ions.

To address this crucial limitation, we report here a systematic QM/MM study of the impact of all 15 Ln^3+^ ions on the coordination properties and energetics of the enzymatic active site, treating the full protein environment explicitly. Changes in the lanthanide coordination environment are of particular mechanistic interest, as shifts in coordination number could modulate substrate binding and activation, as well as PQQ cofactor reactivity.

## Computational Details

### Molecular Dynamics (MD) Simulations

The initial Enzyme–Substrate (ES) complex was constructed from the X-ray PDB structure 4MAE (resolution of 1.6 Å).^19^ This structure presents an α_2_ homodimer, the α subunit is a superbarrel made up of eight four-stranded antiparallel twisted β sheets (W-shaped) that stack radially around a pseudo-8-fold symmetry axis going through the center of the subunit, forming an 8-bladed propeller-like superbarrel. The X-ray structure contains the homodimeric enzyme complexed with a poly­(ethylene glycol) (15P) inhibitor in each active site. The inhibitor was truncated to methanol and it was oriented in such manner that it is consistent with the first step of the two proposed catalytic mechanisms.^1^ That is, in a hydrogen-bond (HB) with Asp299 residue (Figures 1 and 2). Only one protomer of the enzyme was considered for this work. Although the dimeric form is thought to be the functional state *in vivo*,^19^ the active site is not located near the interprotein interface but rather at the center of the β-propeller protein motif. Therefore, considering just one protomer in the simulation box is sufficient to study the effect of metal substitution and its coordination properties on the active site.

**Figure 1:**
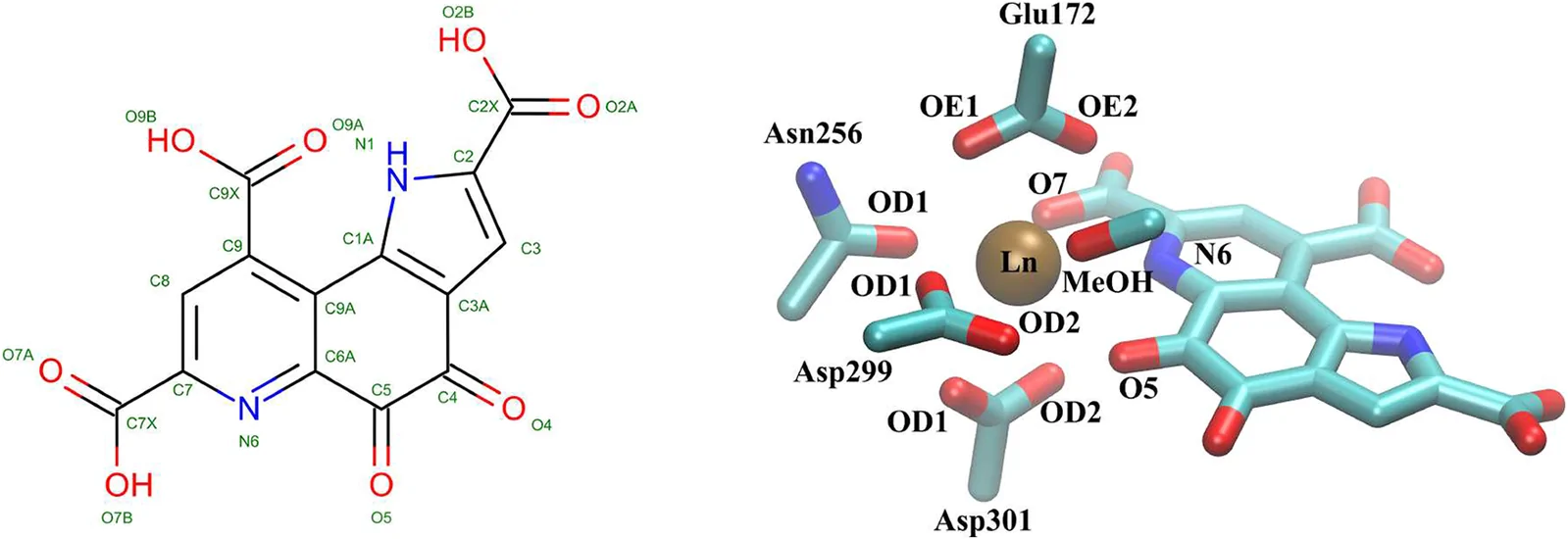
PQQ (left) and Ln^3+^ first coordination shell (right) with their respective numbering schemes.

**Figure 2:**
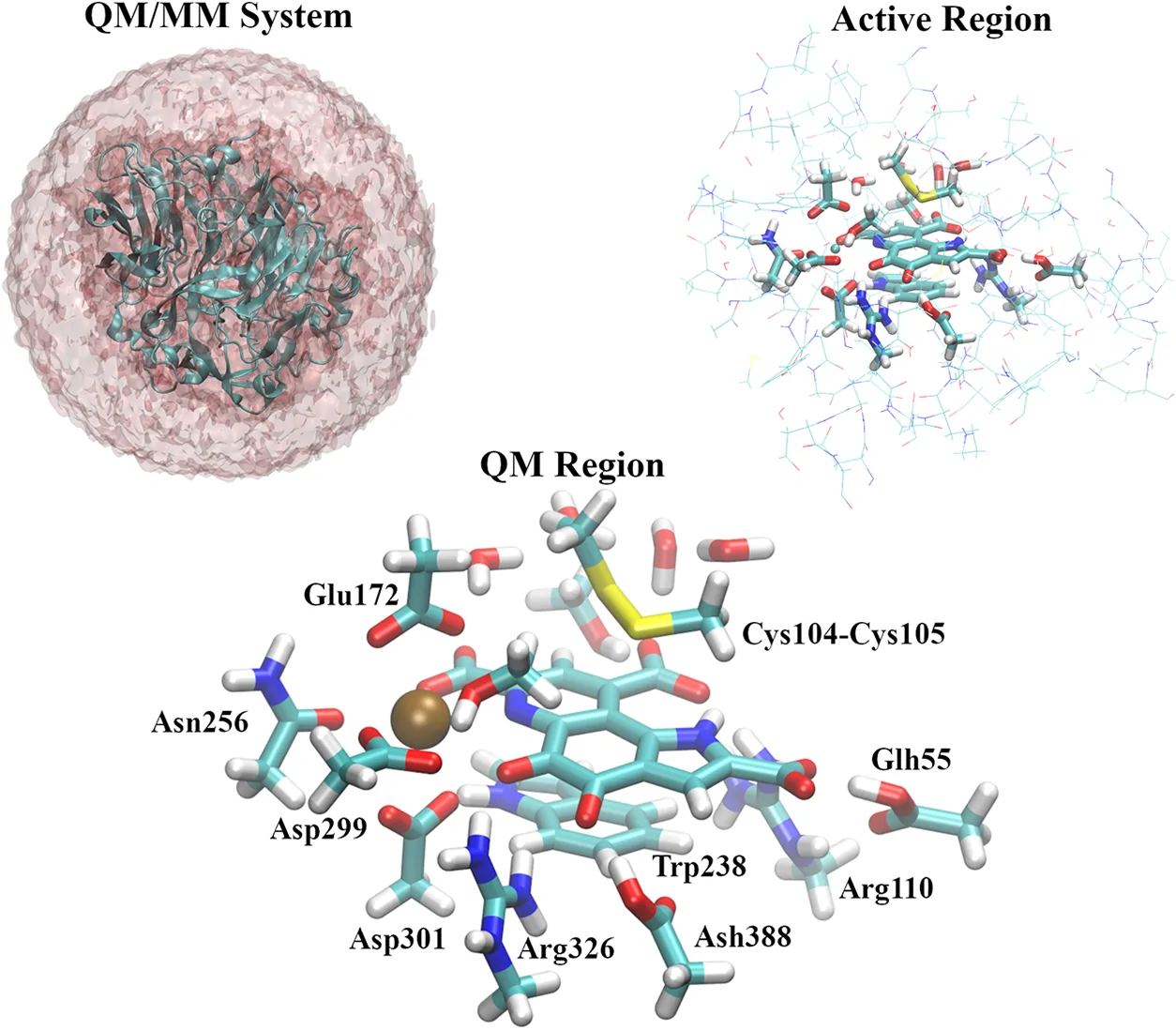
QM/MM system setup: Representative snapshot from the MD simulation with a 42 Å water shell. QM/MM geometry optimization setup: The QM region utilized in the QM/MM geometry optimizations (***R***_opt_) consisted of side chain moieties of Glh55, Arg110, Glu172, Asn256, Asp299, Asp301, Arg326, Cys104-Cys105,Ser169, Trp238 as well as four water molecules near the disulfide bridge and interacting with the PQQ cofactor. Additional details are explained in the text.

The crystal structure reveals that the Ln^3+^ ions are coordinated by four amino acid residues and a PQQ cofactor. The PQQ cofactor is sandwiched between a disulfide bridge (C104–C105) and a TRP residue (W238). Although vicinal disulfides are rare in most proteins, they are common on this type of alcohol dehydrogenases.^20^ Furthermore, the PQQ cofactor is stabilized by an extended HB network and it is not covalenty bound to the protein.

The PQQ cofactor is composed of three coplanar rings. In addition to the axial interactions mentioned above (TRP388 and with the disulfide bridge), many amino acid residues establish equatorial interactions. The Unkefer^21^ numeration scheme of the PQQ cofactor is shown in Figure 1.

To determine the ionization state of MDH’s titratable residues at physiological pH 7.4,^11,22^ the H++ method^23,24^ was used, through its online interface. The results obtained were compared with those generated by PROPKA3.1,^25^ PyPKA,^26^ and DeepKa.^27,28^ The different methods yielded consistent results for the majority of the residues, indicating that they are in their usual protonation states (data not shown). For the residues near the active site, they were manually reviewed and the protonation states were imposed based on the local environment and the first step of the proposed reaction mechanisms (see SI, Section S1). Except for Glu55 and Asp388, all residues retained their standard protonation states. Glu55 and Asp388 were kept protonated because they form hydrogen bonds that stabilize the PQQ cofactor. PQQ was modeled with its three carboxyl groups deprotonated, giving it a net charge of −3. Preliminary MD simulations showed that deprotonating Asp388 and Glu55 destabilized the active site architecture. A stable model was achieved only when both residues were protonated, together with the −3 charge of PQQ. All subsequent simulations were therefore performed with Asp388 and Glu55 protonated.

The classical MD simulations were performed with GROMACS version 2024.3 package.^29,30^ The setup of the system, generation of topology files and solvation of the enzyme, was carried out with CHARMM-GUI web server.^31^ The Amber ff19SB force field^32,33^ was used for all 8828 protein atoms. For the Ln^3+^ ions, (La–Lu, except Pm^3+^, Ho^3+^ and Yb^3+^) the nonbonded model with the Lennard-Jones (LJ) parameters from Li et al.^34^ was employed. For Pm^3+^, Ho^3+^ and Yb^3+^ the LJ parameters of Valentina Migliorati and collaborators^35^ were utilized, respectively combined with the TIP3P water model utilized here. The PQQ cofactor and the substrate were parametrized with Antechamber,^36^ using the GAFF2 force field and AM1-BCC^37,38^ charges. All crystallographic water molecules were retained during the system construction. The enzyme-susbtrate complex was solvated in a cubic periodic box of TIP3P water molecules with any protein atom being at least 10 Å from the edge of the simulation box with a total of 21,472 water molecules. The system was then neutralized modeling a salt concentration of 0.15 M of KCl, resulting in 61 and 60, potassium and chloride ions, respectively.

The MD simulation protocol started with a minimization using the steepest descent algorithm with a convergence criterion of 1000 kJ mol^–1^ nm^–1^. Subsequently, a 125 ps NVT equilibration was performed, restraining the backbone atoms with a force constant of 400 kJ mol^–1^ nm^–2^ and the side chain atoms with 40 kJ mol^–1^ nm^–2^. The temperature was maintained at 320 K using a velocity rescaling thermostat,^39^ following a 125 ps NPT equilibration phase with the same restraints on the protein atoms. Finally, a 50 ns NPT production phase was carried out, removing all side chain and backbone restraints. For the ES complex, specific harmonic restraints were applied to the substrate to maintain its position within the active site (see SI, Section S1). These harmonic restraints were also applied during the NVT and NPT equilibration phases. The velocity rescaling thermostat was used to maintain the temperature at 320 K for both the NVT and NPT phases. This temperature was chosen as it represents a good compromise between stability and reactivity in the kinetic assays of MDHs.^11,22^ Pressure was maintained at 1.0 atm using a stochastic cell-rescaling barostat^40^ with a coupling constant of 5.0 ps and a compressibility of 4.5 × 10^–5^ bar^–1^. Nonbonded interactions were treated with a 0.9 nm cutoff, long-range electrostatics were calculated using the Particle Mesh Ewald (PME)^41,42^ method, and all bonds involving hydrogen atoms were constrained with the LINCS^43,44^ algorithm, allowing for a 2 fs integration time step.

Three replicas of MD simulation were carried out for the ES system in the NPT ensemble at 1 atm and 320 K.

### Analysis of the MD Trajectories

The small RMSD obtained in classical trajectories (Figure S1) is consistent with the experimental finding that lighter lanthanides confer both high catalytic activity and thermal stability to the active sites of PQQ-dependent proteins, allowing them to remain stable and active under elevated temperatures (50 °C to 60 °C) that denature alternative calcium-dependent systems,^45,46^ and supports the reliability of the procedure described in the following paragraphs.

To obtain a representative structure from the 50 ns classical MD trajectory, a clustering analysis of the frames was performed using the RMSD of the heavy atoms from the residues near the metal and the PQQ cofactor (see SI Section S1 for details). The Gromos^47^ algorithm implemented in GROMACS was used with an RMSD cutoff threshold of 0.43 Å over the last 20 ns of the MD trajectory (see SI, Section S2). At first sight, a 50 ns sampling might seem insufficient; nevertheless, we emphasize here that our goal is only to obtain an enzyme–substrate complex consistent with the physiological conditions for further QM/MM geometry optimizations.

The characteristic MD snapshot was taken from a fully solvated periodic box and reduced to a spherical solvent region.^48^ This sphere was generated from the simulation box by removing water molecules located more than 42 Å from the center of mass of the protein. This radius ensures that the residue farthest from the center of mass is surrounded by a water layer of at least 4 Å. Furthermore, the K^+^ counterion closest to the protein was retained to ensure a neutral system. Finally, a geometry optimization at the MM level was performed to relax the nanodroplet and avoid structural problems arising from an abrupt cutoff when extracting it from the cubic box.

This procedure was carried out for the trajectory of the holoenzyme (i.e., the protomer with the substrate) Ce^3+^-MDH-ES. To obtain the Ln^3+^-MDH complexes (Ln = La - Lu), the Ln^3+^ ion in the Ce^3+^-MDH complex was substituted, thus generating 15 Ln^3+^-MDH substrate-enzyme systems. Subsequently, each system was processed with the *tleap* module to generate the AMBER topology using the same parameters as before, and with this topology, the multiscale QM/MM calculations in ORCA 6.1.0 were carried out. Additionally, 15 Ln^3+^-MDH systems without the substrate in the active site were also prepared in a similar fashion by eliminating the substrate from the ES complex.

### QM/MM Computations

#### Geometry Optimizations

All LC-ECP QM/MM geometry optimizations and single-point computations were performed using the ORCA software (version 6.1.0),^49,50^ while the all-electron X2C QM/MM single-point computations utilized the ORCA 6.0.1 software version. For the geometry optimizations, the QM region included key residues that stabilize the PQQ cofactor as well as the primary coordination sphere of the Ln^3+^ cation. Additionally, four water molecules near the disulfide bridge that interact with PQQ are included. Overall, the QM region ***R***_opt_ comprises 12 amino acid residues and four water molecules (see Figure 2). Accurately modeling this enzymatic system is a truly challenging task due to the structure of the PQQ cofactor. It is highly extended (spanning ∼10 Å from tail to head) and features three carboxylate groups. The active region (QM region + free to move MM atoms) was obtained with the automatic fragmentation procedure of ORCA, by extending the QM region to include protein backbone and side chain fragments as well as water molecules around 5 Å around any QM atom. The active and QM regions are shown in Figure 2.

For the geometry optimizations, we employed the range-separated hybrid, generalized gradient approximation (GGA) ωB97X-V^51^ functional along with a def2-SVP^52^ basis set for all protein, water molecules, PQQ and MeOH atoms in the QM region. Dispersion corrections were also included using the revised D4 scheme.^53–55^ For the lanthanides, the 4f in-core effective core potential (LC-ECP) was used along with its associated ECPxxMWB-II basis set, where xx = 46 + *n*, and *n* represents the number of 4f electrons in the ground state electronic configuration of the trivalent lanthanides according to the Aufbau configuration, i.e [Xe]­4*f* ^*n*^ (*n* = 0–14). The RIJCOSX^56,57^ approximation on the Hartree–Fock exchange term was utilized. The def2/J^58^ auxiliary basis set was applied to all atoms except the lanthanides in the QM region. For the lanthanides the auxiliary Coulomb-fitting basis set was generated via *autoaux* ^59^ option of ORCA. All computations were done with default integration grids and tight SCF convergence thresholds. From now on, we will refer to the ωB97X-V functional with the revised D4 parameters as ωB97X-D4rev. The QM/MM geometry optimizations and single-point computations were performed considering the electrostatic embedding as implemented in ORCA.^60^

Valencies at the covalent bonds crossing the QM/MM boundary were saturated using hydrogen link atoms. Details of the position of link atoms are presented in the SI (Section S3).

For the geometric optimization of the Ce^3+^–MDH-ES complex, exploratory tests revealed that both the PBE and PBE0 functionals failed to accurately reproduce the Michaelis–Menten complex geometry. Specifically, PBE resulted in an unstable Michaelis–Menten complex, while PBE0 produced an eclipsed conformation of the CH_3_ group relative to the MeOH substrate’s hydroxyl group. The range-separated hybrid ωB97X-D4rev was necessary to alleviate this substrate positioning issue. Therefore, the ωB97X-D4rev functional was selected for this study. Furthermore, range-separated hybrids have been previously benchmarked for protein structure.^61^ Kulik et al.^62^ have stressed that range-separated hybrid functionals are essential for the description of the QM region of enzymatic systems because pure GGA and hybrid GGA functionals spuriously close the HOMO–LUMO gap. Regarding the basis set, def2-SVP was selected, as modest double-ζ basis sets have been shown to provide reliable relative energetics and structural properties in proteinic systems.^61^

In order to obtain accurate energetics we utilized a larger QM region (***R***_En_) to compute single-point energies at the optimized ωB97X-D4rev/def2-SVP geometries. Furthermore, the larger def2-TZVPP basis set^52^ was utilized for all protein atoms in the larger QM region. As we are not interested in precise spectroscopic or magnetic properties, the LC-ECP and its associated basis set for each Ln metal was utilized. This higher level of theory was further validated by all-electron calculations via scalar relativistic all-electron X2C computations as implemented in ORCA. All-electron relativistic single-point energies were also computed utilizing the X2C^63^ Hamiltonian using the X2C-TZVPPall^64^ basis set for all atoms in the QM region but maintaining the same ωB97X-D4rev XC functional. For these X2C computations, the spin mutiplicity was set according to the ground state electronic configuration of trivalent Lns, i.e., [Xe]­4*f* ^*n*^ (*n* = 0–14).

The larger QM region (***R***_En_) was chosen with an approach suggested by Brandt and Jacob.^65–67^ This consists of Point Charge variation Analysis (PCVA) in combination with protein network centralities. The former approach captures residues with a preponderant electrostatic effect, while the latter captures the nonelectrostatic residues that might be crucial in the energetic description of the active site. This combination of PCVA and protein network centralities can be used to systematically construct atom economical QM regions of enzymatic systems. Details of this QM region are given in the SI (Section S4). The resulting QM region, denoted ***R***_En_, consists of 17 residues and 7 water molecules (Table 1).

**Table 1:** Details of the QM Regions Used in This Work^a^

QM region	no. Residues	no. Wats	no. QM atoms	no. link atoms	QM charge
***R***_opt_	12	4	138	12	–1
***R***_En_	17	7	273	30	–1

a Number of QM atoms correspond to the MeOH-bound active site. Detailed descriptions of these QM regions can be found in the main text and in the SI (section S4).

In biology, metal selectivity depends both on affinity differences and conformational changes.^5^ Thus, in order to complement the structural analysis, the affinities of the enzyme for the lanthanide and for the substrate were computed following these reactions

1 $$\mathrm{Ln}{\left(H_{2}O\right)}_{9}^{3+}+\mathrm{MDH}\to {\mathrm{Ln}}^{3+}‐\mathrm{MDH}+9H_{2}O$$

2 $${\mathrm{Ln}}^{3+}‐\mathrm{MDH}+\mathrm{MeOH}{\left(H_{2}O\right)}_{m}\to +\mathrm{holo}‐\mathrm{MDH}+m\left(H_{2}O\right)$$

where *m* denotes the number of water molecules that hydrate the MeOH molecule.

The relative energies were computed taking Ln = La as a reference value, thus we have

3 $$\Delta E_{{\mathrm{Ln}}^{3+}}=E{\left(\mathrm{noSub}\right)}_{{\mathrm{Ln}}^{3+}}-E{\left(\mathrm{noSub}\right)}_{{\mathrm{La}}^{3+}}+E\left(\mathrm{La}{\left(H_{2}O\right)}_{9}^{3+}\right)-E\left(\mathrm{Ln}{\left(H_{2}O\right)}_{9}^{3+}\right)$$

as a measure of the affinity of the MDH for the lanthanide, and

4 $$\Delta E_{\mathrm{MeOH}}=E{\left(\mathrm{ES}\right)}_{{\mathrm{Ln}}^{3+}}-E{\left(\mathrm{ES}\right)}_{{\mathrm{La}}^{3+}}+E{\left(\mathrm{noSub}\right)}_{{\mathrm{La}}^{3+}}-E{\left(\mathrm{noSub}\right)}_{{\mathrm{Ln}}^{3+}}$$

as a measure of the affinity of the MDH for the MeOH susbtrate. Where $E{\left(\mathrm{ES}\right)}_{{\mathrm{Ln}}^{3+}}$ denotes the QM/MM energy of the enzyme–substrate complex and $E{\left(\mathrm{noSub}\right)}_{{\mathrm{Ln}}^{3+}}$ denotes the QM/MM energy in the absence of MeOH substrate. Potential energy values reported here are QM/MM energies at 0 K without entropic or ZPE contributions.

Taking Ln = La as a reference proves quite handy, as it eliminates the need to compute the energies of apo-MDH, MeOH­(H_2_O)_*m*_ or H_2_O. However, the energies of the Ln­(H_2_O)_9_^3+^ complexes are still required. The geometries of these nona-coordinated lanthanides were optimized at the LC-ECP/ωB97x-D4rev/def2-SVP level of theory. Additionally, single-point calculations were carried out at the LC-ECP/ωB97x-D4rev/def2-TZVPP and X2C/ωB97x-D4rev/X2C-TZVPPall levels of theory for the energetic analysis. These single-point computations of the Ln­(H_2_O)_9_^3+^ complexes were carried out applying the SMD continuum solvation model^68^ with water as the solvent, while the geometries were optimized in vacuum.

#### QM/MM MD Simulations

As the optimized geometries only represent a point in every Ln^3+^–MDH-ES complex’s potential energy surface (PES), and the thermal effects might change the observed tendencies in Lns’ coordination in the minimized enzymatic environment, we expanded the description of the 15 Ln^3+^–MDH-ES complexes studied in this work with 2 ps QM/MM MD simulations at the LC-ECP/ωB97x-D4rev/def2-SVP/FF14SB level of theory. In this approach, despite the evident limitations in the sampling of the conformational space of the enzymatic complexes, it is possible to observe the most frequent changes in the coordination sphere of each Ln^3+^ cation, from La to Lu, and describe their population of states with different coordination numbers.

Previous to the QM/MM MD calculations, we performed classical MD simulations using the *pmemd* program of the Amber24 suite^69^ on the Ce^3+^–MDH-ES complex system to minimize, equilibrate, sample the conformational space and select a representative structure from the production stage. First, we generated the MD inputs in the *tleap* program from Amber24 suite^69^ by updating the previous generated parameters for the optimization tasks, to change the protein force field ff19SB to ff14SB. This last protein force field was selected for two main reasons: (1) in the Amber manual it is recommended to combine the ff19SB force field with the four-point polarizable OPC waters for the MD simulations,^69^ which showed to be problematic during our preliminary QM/MM simulations with ORCA; and (2) the combination of the ff14SB force field for protein and TIP3P for waters showed to be compatible with ORCA and notably less computationally expensive than the ff19SB-OPC formula in our tests on the classical MD simulations. The force field parameters of the PQQ cofactor, MeOH substrate and Ln^3+^ ions are identical as the ones defined for the nanosolvated system.

Once generated the MD inputs, the simulation protocol started with ten thousand minimization steps combining the steepest descent and conjugated gradients algorithms, as implemented in the *pmemd* program form the Amber24 suite.^69,70^ Then, the minimized system was heated to 320 K at constant volume and equilibrated in the NVT ensemble for 500 ps, followed by 30 ns in the NPT ensemble. During the NVT equilibration, all the solute heavy atoms were constrained, allowing the solvent to move while the ES complex maintained stable. For the NPT equilibration, we only constrained the protein backbone, cation, PQQ, and substrate. Once the system was equilibrated, we performed the productive MD simulations for 1 μs of simulation time, while restraining the distance from substrate’s OH group to Ce to 3.0 Å. During these simulations, we applied periodic boundary conditions (PBC) with a cutoff of 9 Å for the nonbonded interactions in the direct space, combined with the use of the PME method, as implemented in the *pmemd* program^70^ to compute the long-range electrostatic interactions.

The selected frame from the trajectory was the input for 100 ps of the QM/MM equilibration in the sander program using its interface with the XTB code^71^ to use the semiempirical Hamiltonian GFN2-xTB^72^ to describe the QM region. In this implementation, Ewald electrostatics are incorporated directly into the self-consistent field (SCF) procedure, enabling linear-scaling QM/MM PME calculations. These employed a 1 Å reciprocal-space grid, tinfoil boundary conditions, and a 9 Å direct space cutoff. The equilibrated system at the GFN2-xTB level was the input for the DFT-based 2 ps QM/MM simulations in sander using its interface with ORCA 6.1.1.^73^ As the sander/ORCA external interface does not allow the use of PBC for the QM region, we increased the direct space cutoff for the QM partition to 15 Å, while maintaining the 9 Å cutoff and PBC for the MM partition. On the other, hand, for these simulations, we rationalized the computational costs of the initial simulations by replacing the Ce^3+^ cation in the topology for each Ln^3+^ using the *parmed* program from the Amber24 suite,^69^ to generate the MD inputs. In this sense, to make viable the DFT-based QM/MM MD simulations on the ES complex for all Lns, the QM region was set by excluding the water molecules and the two cysteine residues originally in the QM region for the optimizations in ORCA (see Figure 2).

The coordination sphere of each Ln^3+^ cation was characterized from the QM/MM MD trajectories using a geometric criterion. A donor atom was considered to belong to the first coordination shell when its distance to the metal was equal to or shorter than 3.1 Å. For the carboxylate ligands (Glu172, Asp299 and Asp301) an additional symmetry condition was imposed in order to distinguish genuine chelation from a strongly asymmetric contact: a residue was counted as bidentate only when both carboxylate oxygens lay within the 3.1 Å cutoff and the difference between the two Ln–O distances did not exceed 0.5 Å; otherwise the residue was counted as monodentate. The PQQ cofactor was assigned a denticity between zero and three according to the number of its O5, N6 and O7A atoms inside the cutoff, whereas Asn256 and the methanol substrate can only contribute a single donor atom each. The total coordination number reported here is therefore the sum of the contributions of the six intrinsic ligands of the active site, and its maximum attainable value is 11. The first 100 frames of every trajectory were discarded as equilibration, and the populations were obtained from the remaining frames of each 2 ps trajectory.

### The Goal of This Work

In this work, we report the impact of Ln^3+^ ion on the coordination properties of the active site. The structure of the Ce^3+^-MDH Michaelis–Menten complex was determined with MD simulations. This Ce^3+^-MDH equilibrated structure was utilized to construct a nanosolvated ES complex the metal center was substitued to obtain 15 Ln^3+^-MDH-ES (Ln= La–Lu) complexes. Furthermore the substrate was removed to obtain additional 15 complexes without the substrate. These complexes were subjected to electronic structure geometry optimizations at the ωB97X-D4rev/def2-SVP/FF19SB level of theory. From these optimized geometries, the energetic analysis was carried out by computing the affinities of eqs 3 and 4.

From the experimental side, reports of Ln-MDHs include La, Ce, Nd or Eu in the active site. These structures reveal a high degree of structural conservation between the active sites, with the first coordination shell being nona-coordinated by two ASP (one bidentate and one monodentate), a GLU (bidentate), an ASN (monodentate) and PQQ (tridentate). If we include the substrate, which in some structures is also found coordinated to the metal center, an overall coordination number of 10 is found.

As the metal center gets smaller, the ligand–ligand repulsion will expel one or more ligands from the coordination shell. Here we expand our understanding of which ligands change denticity along the series and how this effect might impact the structure of the active site.

## Results

### QM/MM Optimized Geometries

We addressed here how the metal substitution affects the geometry of the active site of MDH with and without presence of the substrate, and how this could relate to the biological preference of MDH for early lanthanides.

The evolution of the first-shell Ln-ligand distances along the series is shown in Figures 3 and 4. All distances are presented in Tables S2–S5 of the SI. The numbering scheme of the ligands in Ln^3+^ first coordination shell is defined in Figure 1.

**Figure 3:**
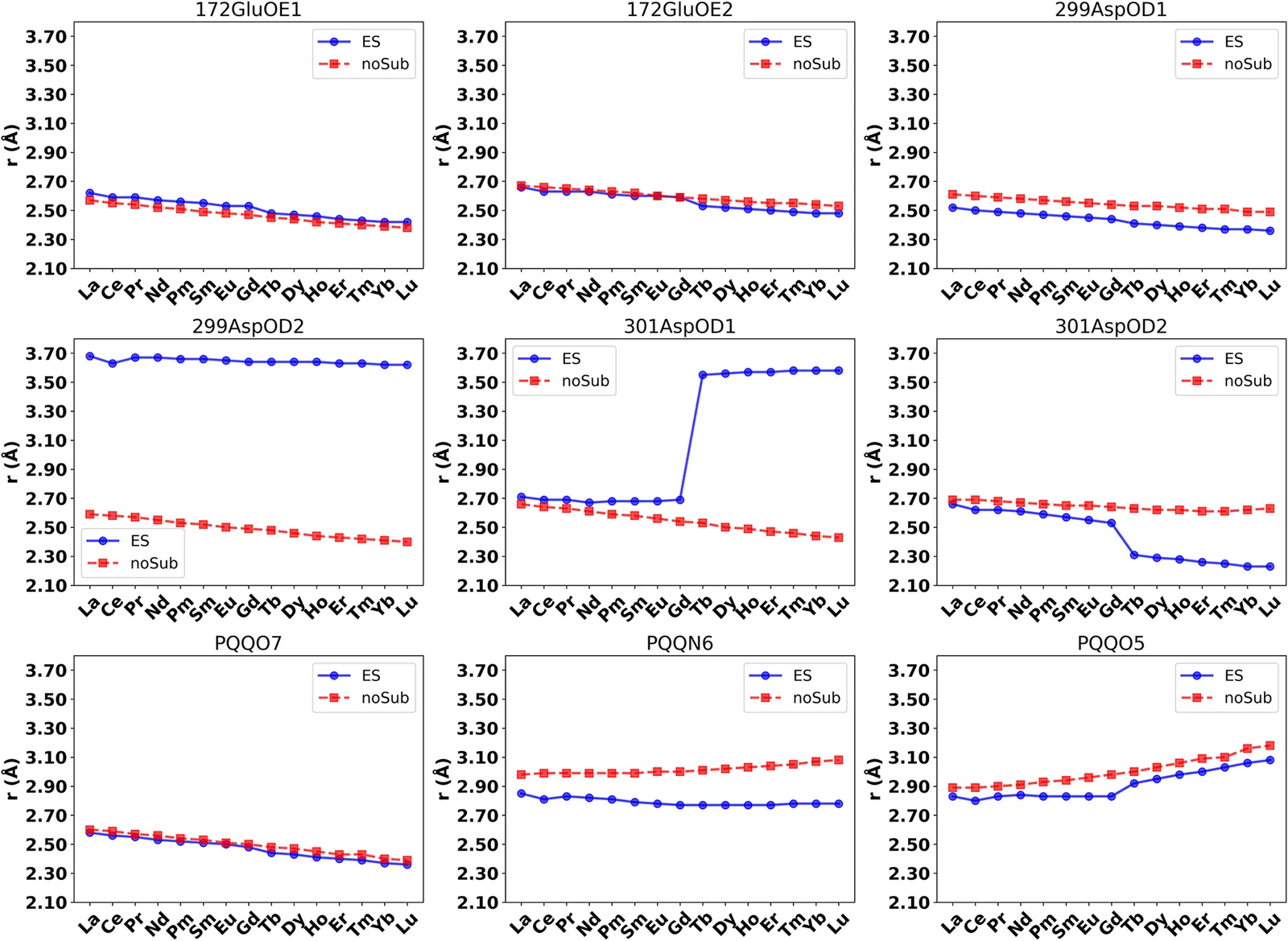
Comparison of lanthanide coordination distances in Ln^3+^-MDH. Values computed at the ωB97X-D4rev/def2-SVP/FF19SB level of theory. ES denotes the Enzyme–Substrate complex; noSub denotes the substrate-free Enzyme. For the atom naming scheme, refer to Figure 1.

**Figure 4:**
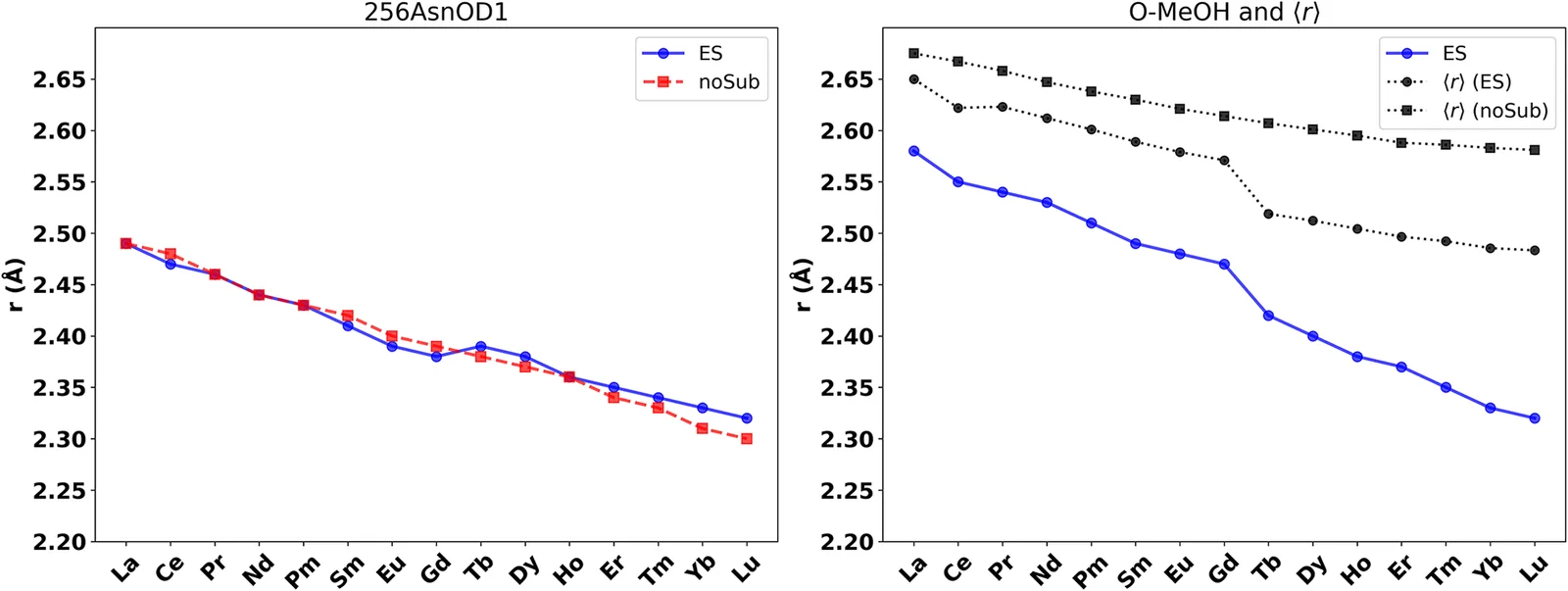
Comparison of lanthanide coordination distances to specific residues in Ln^3+^-MDH. Distances shown are to the coordinating oxygen atoms of residue Asn256 and bound methanol (MeOH). Values computed at the ωB97X-D4rev/def2-SVP/FF19SB level of theory. For reference, the MeOH binding distance is contrasted against the average distance to all other coordinated atoms, denoted as ⟨*r*⟩. For the atom naming scheme, refer to Figure 1.

In the substrate-bound form, the active site displays a coordination sphere that undergoes a clear transition near the middle of the series. While residues Glu172 and Asn256, along with the PQQ O7 atom, exhibit the expected monotonic decrease in bond distances characteristic of the lanthanide contraction, other ligands show a different behavior. Notably, Asp299 remains monodentate across the entire series, with the Ln–OD2 distance consistently exceeding 3.6 Å. This contrasts sharply with the substrate-free form, where Asp299 coordinates in a bidentate fashion for all lanthanides, with both OD1 and OD2 distances contracting smoothly from 2.61 Å and 2.59 Å in La to 2.49 Å and 2.40 Å in Lu, respectively. This indicates that the presence of the substrate actively displaces one of the Asp299 carboxylate oxygens, likely to accommodate the methanol ligand and the PQQ cofactor within the coordination environment.

The most pronounced effect of substrate binding is observed for Asp301. In the absence of substrate, Asp301 acts as a stable bidentate ligand throughout the entire lanthanide series, with both Ln–OD1 and Ln–OD2 distances decreasing steadily from 2.66–2.69 Å in La to 2.43–2.63 Å in Lu. However, upon substrate binding, Asp301 undergoes a clear transition from bidentate to monodentate coordination that initiates at Tb. For the larger ions (La–Gd), Asp301 remains bidentate with Ln–OD1 and Ln–OD2 distances of *ca*. 2.70 Å and 2.53–2.66 Å, respectively. From Tb onward, the OD1 atom is decisively excluded from the first coordination sphere, with its distance over 3.55 Å, while the OD2 distance shortens considerably to 2.23–2.31 Å to compensate.

Interestingly enough, this aspartate residue, which is conserved in Ln-dependent MDH and Alcohol dehydrogenase (ADH) enzymes, has been shown to be needed for *in vivo* catalityc function, *in vivo* activity, and La^3+^ coordination.^9^ That is, this Ln^3+^-coordinating aspartate is essential for the enzymatic function of the MDH enzyme. This shift in coordination mode might give insight into the biological preference of MDH for the early lanthanides.

The coordination distances to the PQQ cofactor are critically dependent on both the substrate and the ionic radius. In the substrate-bound enzyme, PQQ clearly acts as a tridentate ligand for the early lanthanides (La–Gd), with O7, N6, and O5 all within bonding distance. The Ln–O5 distance remains stable at 2.83 Å for these ions but then increases steadily from Tb onward, reaching 3.08 Å for Lu. The Ln–N6 distance, however, remains remarkably constant (2.7–2.78 Å) for Tb–Lu. In the substrate-free form, the PQQ interaction is notably weaker. The Ln–N6 distances are significantly longer (2.98 Å in La, increasing to 3.08 Å in Lu), and the Ln–O5 distances start higher (2.89 Å) and increase monotonically without an initial plateau. Nevertheless, the coordination shell of the Ln^3+^ ions in this enzymatic environment may be highly labile, as observed in aqueous solution.^74,75^ In particular, since the O5- and N6-PQQ ligands are polar in nature rather than hard carboxylate ligands such as those of ASPs and GLUs, it can be expected that their interactions with the metal are weaker, consistent with the larger observed distances. Furthermore, C5-PQQ is highly reactive in isolated PQQ^76^ and this carbonyl moiety may be involved in either the addition–elimination mechanism or the hydride transfer mechanism.^1^ Thus, these ligands may require a larger separation from the lanthanide for the reaction to occur. In addition, the potential dynamic character of this coordination shell along the reactive path will be explored in the near future by some of us at the GFN2-xTB/MM MD level, following a reparametrization of the semiempirical GFN2-xTB method to accurately model this active site. This will shed light on the coordination mode of the PQQ cofactor from a dynamic point of view. However, this issue is out of the scope of the present work.

We consider the PQQ coordination mode to be tridentate throughout the series, although the binding becomes progressively more distorted for the heavier lanthanides, as evidenced by the steady elongation of the Ln–O5 distance from Tb onward. The only ligand clearly expelled from the coordination shell is Asp301-OD1 in the substrate-bound complexes, leading to CN = 10 for La–Gd and CN = 9 for Tb–Lu, while in the substrate-free form the coordination number remains unchanged along the series. This demonstrates that the substrate is essential for drawing PQQ into a tight, well-defined tridentate binding mode, an arrangement that is optimal for lighter lanthanides but becomes increasingly strained as the ionic radius decreases toward Lu.

Average Ln–ligand distances decrease across the series, confirming that the lanthanide contraction persists within the enzymatic environment. Now, focusing only on the ES complexes. The lanthanide center as lewis acid is in charge of binding the substrate. The Ln-O­(MeOH) distance gets shorter with decreasing ionic radii of the metal center (Figure 4 and SI tables S2–S3) from 2.58 Å with La to 2.32 Å with Lu, following a decreasing trend with increasing atomic number. This confirms its role as a Lewis acid.

In summary, the presence of the substrate does not merely occupy a coordination site; it restructures the active site, making it more prone to steric crowding. The decoordination of Asp301-OD1, occurring precisely at the Tb/Gd divide, represents a rearrangement of the first coordination shell that is fully dependent on substrate binding. This transition is a prime structural candidate for explaining the enzymatic preference for lighter lanthanides, as the altered geometry for the heavier ions is likely incompatible with efficient catalysis. The crystal structure of the Ce-bound enzyme shows a distance trend (Ce–O5 = 2.55 Å, Ce–N6 = 2.80 Å, Ce–O7 = 2.70 Å) that differs from our optimized values (Ce–O5 = 2.80 Å, Ce–N6 = 2.81 Å, Ce–O7 = 2.56 Å), which may reflect the different physical state (e.g., ensemble averaging in the crystal vs a 0 K minimum) or a different redox state of the PQQ cofactor, as has been previously proposed.^19^

It is well-known that the coordination number decreases along the Ln series both in solution^75,77^ and in solid state;^78^ however, in this more intricate enzymatic environment the coordination structure has not been extensively studied.

Our computational results are in good agreement with the DFT cluster model of Daumann et al.,^11,12^ who observed a change in Asp301 coordination from bidentate to monodentate; while, PQQ remained tridentate throughout the series with increasing metal–ligand distances. In contrast, our results do not support the coordination shift from tridentate-to-unidentate PQQ reported by Tsushima^18^ from MD simulations in the absence of substrate. We attribute this discrepancy to two possible factors: the absence of substrate in that study, which as shown above is essential for maintaining a tight tridentate PQQ binding mode, and the inherent limitations of classical force fields in accurately describing the complex electrostatic and coordination environment of lanthanide-binding active sites. Furthermore, Tsushima observed an influx of one to two water molecules into the lanthanide’s first coordination shell during the 50 ns MD simulations. This latter effect, however, might be an artifact arising from the protonation states of Asp388 and Glu55, which, as we have argued, should be considered protonated. The work by Tsushima does not explicitly address the protonation states of residues adjacent to the PQQ cofactor’s carboxyl groups. The methodology merely indicates that the protonation state of the protein was set to reflect a physiological pH of 7.4. Consequently, it can be inferred that standard p*K*_a_ values were applied, resulting in the deprotonation of ASP388 and GLU55.

Our systematic DFT optimizations across the full La–Lu series additionally capture the progressive elongation of Ln–O5 and the abrupt decoordination of Asp301-OD1 at Tb within a single framework, offering a more complete series-wide characterization of Ln–ligand distances than either prior study provided. The DFT/MM MD simulations presented in the following section extend this characterization to physiological temperature and show that these trends are preserved when thermal motion is taken into account.

### Coordination Dynamics of the Active Site at 320 K

The geometries discussed above correspond to stationary points on the potential energy surface and therefore describe the active site in the absence of thermal motion. Since the bidentate and monodentate arrangements of Asp301 may be close in energy, and since the vibrational and conformational contributions that determine their relative weight at finite temperature are not captured by a 0 K description, it is pertinent to ask whether the rearrangement identified above survives thermal averaging. To address this question we analyzed the 2 ps DFT/MM MD trajectories computed for the 15 Ln^3+^–MDH-ES complexes at 320 K, which incorporate thermal and entropic effects by construction. The resulting populations are collected in Figure 5, and the corresponding numerical values are given in the SI (Tables S6 and S7).

**Figure 5:**
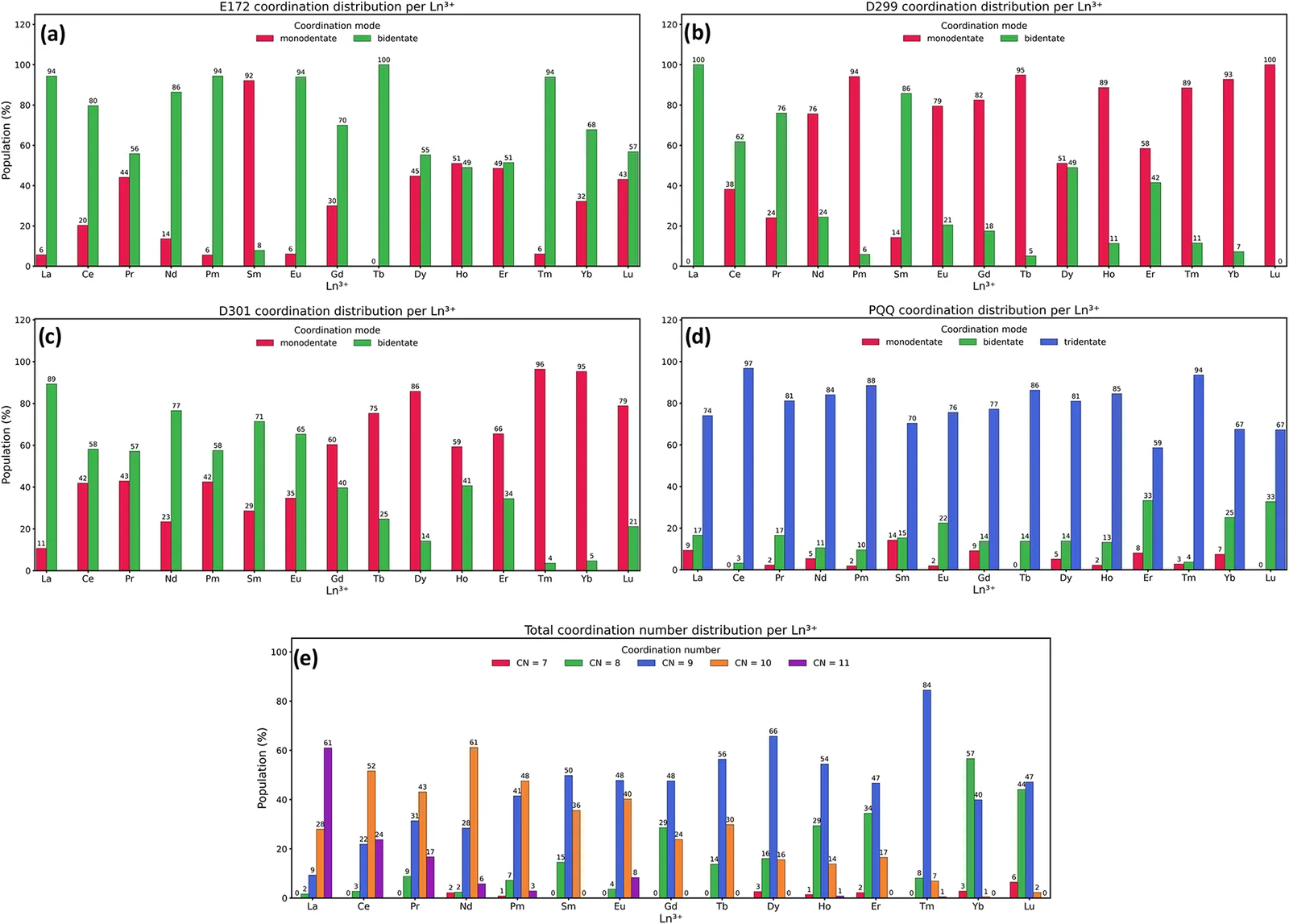
Coordination mode populations obtained from the 2 ps DFT/MM MD simulations of the 15 Ln^3+^–MDH-ES complexes at 320 K, computed at the LC-ECP/ωB97X-D4rev/def2-SVP/FF14SB level of theory. (a) Glu172; (b) Asp299; (c) Asp301; (d) PQQ cofactor; (e) total coordination number of the Ln^3+^ cation.

The behavior of Asp301 at finite temperature reproduces the trend obtained from the optimized geometries (see Figure 5c). The bidentate mode is the majority among the species for the seven lightest cations, with populations of 89% for La, 77% for Nd, 71% for Sm and 65% for Eu. The two modes become comparable at Gd, Ho and Er, for which the monodentate arrangement is sampled near 60% of the time, while the monodentate mode is clearly dominant for Tb, Dy, Tm, Yb and Lu, rising from 75% for Tb to 96% for Tm and 95% for Yb. The transition therefore occurs in the same region of the series as in the optimized structures, namely at the Gd/Tb boundary, but at 320 K it takes the form of a progressive shift in populations rather than an abrupt change. This is the expected outcome for an equilibrium between two accessible coordination modes, and it indicates that the decoordination of Asp301-OD1 is not an artifact of the 0 K treatment: thermal motion does not erase the effect of the lanthanide contraction on this residue, but rather redistributes it over an ensemble.

The total coordination number follows the same direction (see Figure 5e). The most populated value decreases monotonically along the series, from 11 for La to ten for Ce–Pm, nine for Sm–Tm and eight or nine for Yb and Lu. A more compact measure of this trend is the combined population of the states with CN ≥ 10, which decreases from 89% for La and 76% for Ce to 24% for Gd, and finally to 1% and 2% for Yb and Lu, respectively. Conversely, the states with CN ≤ 8, which are essentially unpopulated for the lightest cations, account for 60% of the sampled configurations for Yb and 50% for Lu. The progressive reduction of the coordination number that is well documented for the lanthanide series in aqueous solution^75,77^ and in the solid state^78^ is thus also operative inside the enzymatic active site at physiological temperature.

It is worth noting that the maximum value of 11, which corresponds to Asn256, the methanol substrate, the tridentate PQQ cofactor and the three carboxylate residues acting simultaneously as bidentate ligands, is reached to an appreciable extent only by La (61%) and Ce (24%; see Figure 5). Only the largest cations of the series offer a coordination sphere wide enough to accommodate every available donor atom at its full denticity. Additionally, no water molecule entered the first coordination shell of any Ln^3+^ cation during these simulations. This result reinforces our interpretation of the water influx reported by Tsushima,^18^ probably, as a consequence of the protonation states assigned to Asp388 and Glu55 rather than an intrinsic feature of the active site, since here the shell remains saturated by protein and cofactor donors throughout.

The PQQ cofactor remains tridentate at finite temperature (see Figure 5d). Its tridentate population exceeds 59% for every cation of the series and lies above 70% for 13 of the 15 complexes, reaching 97% for Ce and 94% for Tm. The residual populations correspond to transient bidentate arrangements, which become somewhat more frequent for the heaviest cations (33% for Er and Lu, 25% for Yb), in agreement with the progressive elongation of the Ln–O5 distance found in the optimized geometries. These simulations therefore confirm, now from a dynamic description at the DFT/MM level, that the tridentate binding mode of PQQ is preserved along the whole series in the presence of the substrate, and that the tridentate-to-unidentate shift reported from classical MD in the substrate-free enzyme^18^ is not recovered when the substrate is present and the electronic structure of the active site is treated quantum mechanically.

Glu172 is the most stable of the three carboxylate ligands (see Figure 5a), remaining predominantly bidentate for 14 of the 15 cations, with populations between 49% and 100%. Asp299 is bidentate for the three lightest cations (100% for La, 62% for Ce and 76% for Pr) and becomes predominantly monodentate from Nd onward (see Figure 5b). The single exception to both trends occurs for the same cation: in the Sm complex, Glu172 is monodentate 92% of the time while Asp299 is bidentate 86% of the time (see Figure 5a,b). The two residues are therefore anticorrelated, and the total coordination number is not affected by the exchange, which indicates that Glu172 and Asp299 compete for the same coordination site rather than reflecting an isolated sampling artifact. This competition illustrates the lability of the carboxylate shell in this active site, which is consistent with the behavior observed for lanthanide aqua ions.^74,75^

Taken together, these simulations show that the coordination changes identified from the optimized geometries persist when thermal motion is taken into account. The rearrangement of Asp301 is particularly relevant in this respect, since this residue is conserved in Ln-dependent MDH and ADH enzymes and has been shown to be required for *in vivo* catalytic function and for La^3+^ coordination.^9^ The finite temperature description therefore does not weaken the structural argument developed above; it places it on an ensemble footing, showing that the early lanthanides retain a coordination environment in which Asp301 is predominantly chelating, whereas the heavier cations sample almost exclusively the monodentate arrangement.

The limitations of this analysis should nevertheless be stated explicitly. The trajectories are 2 ps long and a single replica was computed for each cation, so the reported populations should be read as an indication of the most frequently sampled coordination modes rather than as converged ensemble averages. More importantly, these simulations do not provide free energies. Obtaining converged free-energy profiles for these systems (i.e., umbrella sampling and/or metadynamics) is at present prohibitively expensive at the DFT/MM level, while the semiempirical Hamiltonians that would make such sampling affordable describe the lanthanide coordination sphere only approximately. Work addressing this issue through a reparametrization of the GFN2-xTB method for this active site is currently in progress, but it lies outside the scope of the present study.

### Relative MDH Affinities for Substrate and Metal Center

The relative affinities computed with both relativistic approaches, X2C and LC-ECP, are shown in Figure 6. The two methods yield mutually consistent results across the entire lanthanide series, with a maximum absolute deviation of 2.43 kcal/mol observed for Sm. This agreement lies within the expected accuracy of DFT for lanthanide-containing systems, where the intrinsic error of any exchange-correlation functional exceeds the threshold of chemical accuracy (1 kcal/mol) or even 5 kcal/mol, which has been suggested as the chemical accuracy for lanthanide systems.^79^ Both treatments of scalar relativistic effects can therefore be considered equivalent for the purposes of this analysis. All relative affinities are presented in the SI (Table S8).

**Figure 6:**
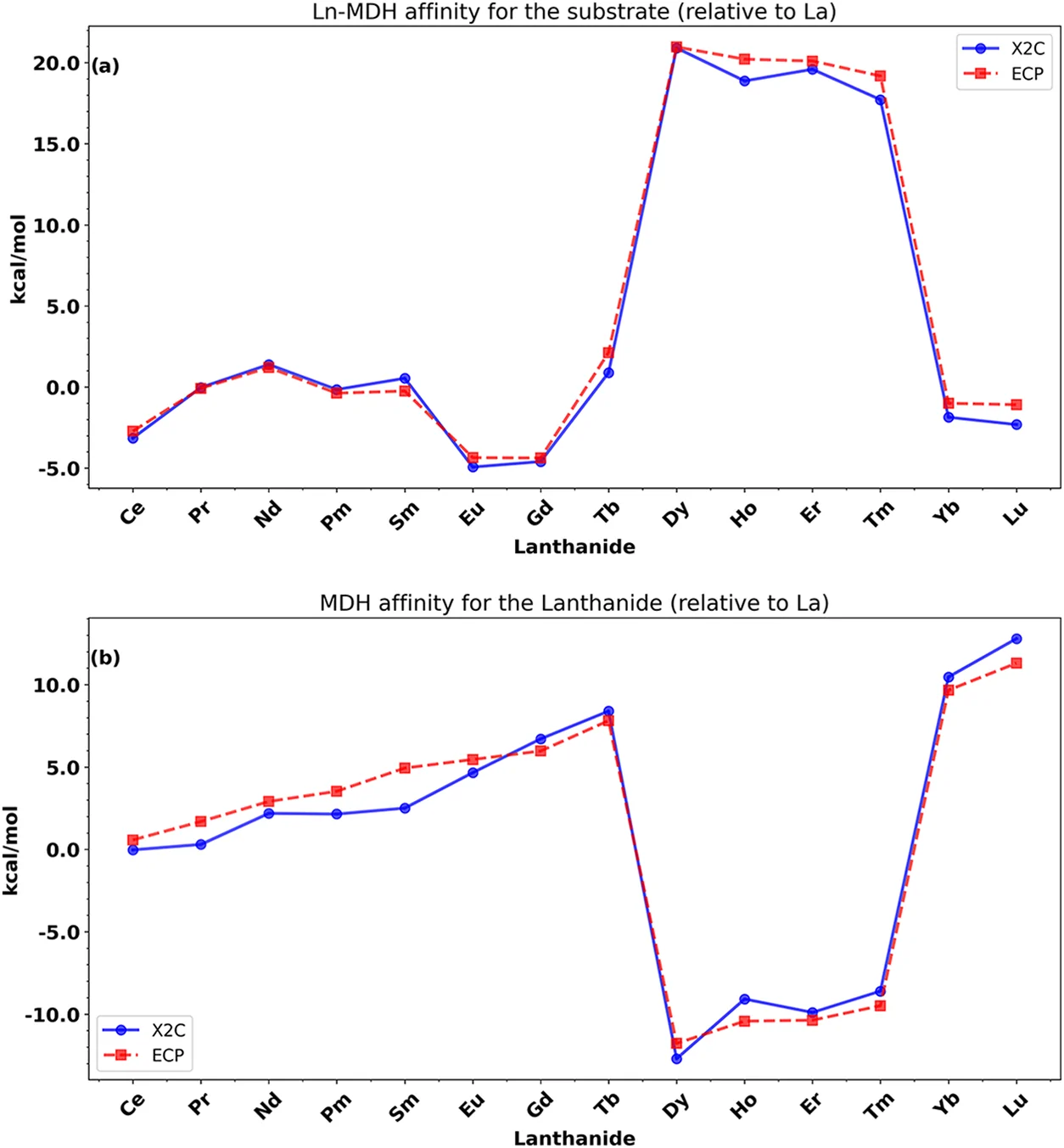
(A) Relative Ln-MDH affinity for MeOH. (B) Relative MDH affinity for Ln.

Figure 6a shows the affinity of MDH for the MeOH susbtrate, computed relative to La, corresponding to the eq 4. The trend is nonmonotonic across the series. The early lanthanides Ce through Pm display affinities near zero (within 5 kcal/mol), indicating that substitution of La by these metals causes negligible perturbation of substrate binding. These metals retain the CN = 10 coordination geometry identified in the structural analysis. Beyond Tb, the trend reverses sharply: Dy through Tm display the most unfavorable substrate affinities of the entire series, reaching +19 to +21 kcal/mol relative to La. This pronounced destabilization coincides with the NC = 10 to 9 transition at Tb. Yb and Lu recover toward near-zero values, suggesting partial restoration of a favorable coordination environment at the end of the series, consistent with a favorable lower coordination number for the smallest lanthanides.

Figure 6b shows the affinity of MDH binding site for each lanthanide ion, computed relative to La for the metal-substituted enzyme in the absence of substrate, corresponding to eq 3. The early to mid lanthanides Ce through Gd show a gradual increase in relative energy, indicating progressively less favorable enzyme binding compared to La as the series is traversed. A sharp reversal occurs at Dy, where both methods yield values of −9 to −13 kcal/mol, identifying Dy, Ho, and Er as the lanthanides that bind to the enzyme most favorably across the series. Recall that these Tb–Lu structures have Asp301 as a monodentate ligand, which might be a nonreactive state of the enzyme. Yb and Lu return to nonfavorable values of +9 to +13 kcal/mol, indicating that the smallest lanthanides are no longer accommodated favorably within the binding site.

Although the MDH will bind Dy–Er preferably, these Ln-MDH complexes do not support a favorable binding of the MeOH substrate. This inverse trend suggests that the biological selection of early Lns reflects a balance between these two competing geometric requirements rather than the maximization of either interaction alone.

Taken together, the two panels in Figure 6 reveal a clear decoupling between both affinities across the lanthanide series. Nevertheless, focusing on the La–Gd window, the ones that are biochemically relevant, we found that MDH affinity for lanthanides is enhanced with earlier lanthanides while showing a pretty similar affinity for the MeOH substrate (within 5 kcal/mol). Interestingly, this could explain the biological preference of MDH for the early lanthanides. This is so because within this La–Gd window the binding of the early lanthanides is preferred over that of the heavier ones.

This is another example that shows the relevance of the coordination properties of cations in the preference of biological structures, as in the case of ion-selective channels.^80,81^

Friedman^82^ reported that in a vacuum cluster model of Ln^3+^-MDH (in the absence of substrate), affinity for the active site increases progressively with the atomic mass of the lanthanide (Ln = La, Sm, Gd, Dy and Lu). In contrast, we observe a nonmonotonic trend that deviates sharply from Friedman’s results. Specifically, we find the exact opposite behavior for the early series (Ce–Tb), enhanced affinity for the intermediate series (Dy–Tm), and the weakest affinity relative to La for the heaviest ions (Yb and Lu). This divergence suggests that the protein environment plays a key role on the energetic properties of the active site.

However, it should be noted that our energetic quantities emerge from the Ln coordination properties on the active site in the Michaelis–Menten complex and do not directly account for the full determinants of enzymatic activity, which additionally might involve the redox cycling efficiency of PQQ and the kinetics of substrate and product exchange. The relationship between the computed energies reported here and the experimentally observed Ln-dependent activity of MDH therefore warrants further investigation.

To strengthen the conclusion on the relevance of the coordination properties of cations in the enzyme’s preference for the lighter Lns, due almost exclusively to their larger size and high coordination numbers, further calculations were performed with Y^3+^; this rare-earth element belongs to the same chemical group as La^3+^, but it is lighter and smaller, and its coordination properties are similar to the heavier Lns. The configuration of the active site and its affinity for Y^3+^ turned out to be very similar to those of Er^3+^ and Tm^3+^, as well as the affinity of the ion-coordinated site for MeOH. The numerical data can be found in SI (Tables S9 and S10).

## Conclusions

We have presented a systematic QM/MM characterization of the Ln^3+^-MDH active site across the full lanthanide series, considering the protonated states of Glu55 and Asp388, which are essential for a realistic description of the coordination environment. Geometry optimizations of the 30 Ln^3+^-MDH complexes (ES and substrate-free) reveal that the lanthanide contraction persists within the enzymatic environment, while the substrate plays an active role in restructuring the first coordination sphere. In the Michaelis complex, the increased steric crowding imposed by methanol binding triggers a bidentate-to-monodentate transition of Asp301 at the Gd/Tb boundary, reducing the coordination number from 10 to 9. This shift in Asp301 coordination mode might give insight into the biological preference of MDH for the early lanthanides. The 2 ps DFT/MM MD simulations performed for the 15 complexes at 320 K show that this rearrangement is preserved at physiological temperature: Asp301 is predominantly bidentate for the lightest cations, both modes become comparable at Gd, Ho and Er, and the monodentate mode dominates for Tb, Dy, Tm, Yb and Lu, while the most populated coordination number decreases along the series and the PQQ cofactor remains tridentate throughout.

Energetic analysis reveals a decoupling between substrate and metal binding affinities. Nevertheless, focusing on the biologically relevant window (La–Gd), the MDH affinity for the metal center is enhanced with the early lanthanides, while the substrate affinity remains almost constant. These results contrast with previous vacuum cluster models and underscore the importance of the full protein environment in determining both the structural and energetic properties of the active site.

Taken together, our results provide a structural rationale for the biological preference of MDH for lighter lanthanides: the 10-coordinate geometry of the early Ln^3+^ ions is compatible with optimal substrate positioning, whereas the coordination rearrangement induced by heavier ions is geometrically incompatible with efficient catalysis. The finite temperature simulations reported here indicate that this rearrangement is a robust structural feature of the active site rather than a property of the optimized geometries alone.

We emphasize that the present work establishes a structural relationship and not a kinetic one. The coordination change described here is offered as one plausible contribution to the observed lanthanide dependence of MDH activity, and it is not mutually exclusive with the other factors that have been proposed in the literature, such as the Lewis acidity of the metal center, the redox properties of the PQQ cofactor, or the kinetics of substrate and product exchange. Establishing the relative weight of these contributions would require the free-energy barriers of the catalytic steps to be computed for the whole series, which is beyond the scope of this study for the reasons discussed above. The results presented here should therefore be read as a detailed structural and dynamic characterization of the Michaelis-Menten complex across the lanthanide series, which constrains the possible explanations of the biological selectivity of MDH without by itself demonstrating a causal link to the measured activities.

## Supplementary Material



## Notes

Details about force-field-based molecular dynamics; ionization states of titratable residues; QM region justification; link-atom positioning; QM/MM 0 K optimal Ln–ligand distances; coordination mode populations from DFT/MM MD simulations; relative affinities; input files for the MD and ORCA QM/MM simulations of the Ce^3+^-MDH enzyme–substrate complex and its substrate-free form (with inputs for the remaining 14 lanthanides readily generatable from deposited files or available upon request) (PDF)

## References

[1] Daumann, L. J. ; Pol, A. ; den Camp, H. J. O. ; Martinez-Gomez, N. C. A Perspective on the Role of Lanthanides in Biology: Discovery, Open Questions and Possible Applications. In Advances in Microbial Physiology; Elsevier, 2022; Vol. 81, pp 1–24. 36167440 10.1016/bs.ampbs.2022.06.001

[2] Tani, A. Lanthanide Utilization by Organisms: An Overview. In Lanthanides in Enzymology and Microbiology; Elsevier, 2025; pp 3–27 10.1016/B978-0-443-13307-7.00001-3.

[3] Yang W., Wu K., Chen H., Huang J., Yu Z. (2024). Emerging role of rare
earth elements in biomolecular functions. ISME
J..

[4] Rocha R. A., Alexandrov K., Scott C. (2024). Rare earth elements in biology: From
biochemical curiosity to solutions for extractive industries. Microb. Biotechnol..

[5] Cotruvo J. A. (2019). The chemistry
of lanthanides in biology: recent discoveries,
emerging principles, and technological applications. ACS Cent. Sci..

[6] Daumann L. J. (2019). Essential
and ubiquitous: the emergence of lanthanide metallobiochemistry. Angew. Chem., Int. Ed..

[7] Picone N., den Camp H. J. O. (2019). Role of rare earth elements in methanol oxidation. Curr. Opin. Chem. Biol..

[8] Chistoserdova L. (2011). Methylotrophy
in a lake: from metagenomics to single-organism physiology. Appl. Environ. Microbiol..

[9] Good N. M., Fellner M., Demirer K., Hu J., Hausinger R. P., Martinez-Gomez N. C. (2020). Lanthanide-dependent alcohol dehydrogenases
require
an essential aspartate residue for metal coordination and enzymatic
function. J. Biol. Chem..

[10] Phi M. T., Singer H., Zäh F., Haisch C., Schneider S., den Camp H. J. O., Daumann L. J. (2024). Assessing
Lanthanide-Dependent Methanol
Dehydrogenase Activity: The Assay Matters. ChemBioChem.

[11] Jahn B., Pol A., Lumpe H., Barends T. R., Dietl A., Hogendoorn C., den Camp H. J. O., Daumann L. J. (2018). Similar
but not the same: first kinetic
and structural analyses of a methanol dehydrogenase containing a europium
ion in the active site. ChemBioChem.

[12] Lumpe H., Pol A., den Camp H. J. O., Daumann L. J. (2018). Impact of the lanthanide
contraction on the activity of a lanthanide-dependent methanol dehydrogenase-a
kinetic and DFT study. Dalton Trans..

[13] Featherston E. R., Rose H. R., McBride M. J., Taylor E. M., Boal A. K., Cotruvo J. A. (2019). Biochemical and structural characterization
of XoxG and XoxJ and their roles in lanthanide-dependent methanol
dehydrogenase activity. ChemBioChem.

[14] Wehrmann M., Billard P., Martin-Meriadec A., Zegeye A., Klebensberger J. (2017). Functional
role of lanthanides in enzymatic activity and transcriptional regulation
of pyrroloquinoline quinone-dependent alcohol dehydrogenases in Pseudomonas
putida KT2440. mBio.

[15] Bogart J. A., Lewis A. J., Schelter E. J. (2015). DFT Study of the Active Site of the
XoxF-Type Natural, Cerium-Dependent Methanol Dehydrogenase Enzyme. Chem. - Eur. J..

[16] Prejanò M., Russo N., Marino T. (2020). How lanthanide ions affect the addition-elimination
step of methanol dehydrogenases. Chem. - Eur.
J..

[17] Wang L., Liu K., Song Z., Do H., Yang L., Wu J., Jiang L., Yu H. (2025). Influence
of coordination number
and ionic radius on metal ion preference and activity of lanthanide-dependent
alcohol dehydrogenase: insights from mutational studies and density
functional theory. Colloids Surf., B.

[18] Tsushima S. (2019). Lanthanide-induced
conformational change of methanol dehydrogenase involving coordination
change of cofactor pyrroloquinoline quinone. Phys. Chem. Chem. Phys..

[19] Pol A., Barends T. R., Dietl A., Khadem A. F., Eygensteyn J., Jetten M. S., den Camp H. J. O. (2014). Rare earth metals are essential for
methanotrophic life in volcanic mudpots. Environ.
Microbiol..

[20] Sarmiento-Pavía P. D., Sosa-Torres M. E. (2021). Bioinorganic insights of the PQQ-dependent alcohol
dehydrogenases. JBIC, J. Biol. Inorg. Chem..

[21] Houck D. R., Hanners J. L., Unkefer C. J., van Kleef M. A., Duine J. A. (1989). PQQ: Biosynthetic studies in Methylobacterium
AM1 and
Hyphomicrobium X using specific13C labeling and NMR. Antonie van Leeuwenhoek.

[22] Le T.-K., Lee Y.-J., Han G. H., Yeom S.-J. (2021). Methanol dehydrogenases
as a key biocatalysts for synthetic methylotrophy. Front. Bioeng. Biotechnol..

[23] Gordon J. C., Myers J. B., Folta T., Shoja V., Heath L. S., Onufriev A. (2005). H++: a server for estimating p K
as and adding missing
hydrogens to macromolecules. Nucleic Acids Res..

[24] Anandakrishnan R., Aguilar B., Onufriev A. V. (2012). H++ 3.0:
automating p K prediction
and the preparation of biomolecular structures for atomistic molecular
modeling and simulations. Nucleic Acids Res..

[25] Olsson M. H. M., Søndergaard C. R., Rostkowski M., Jensen J. H. (2011). PROPKA3: consistent treatment of
internal and surface
residues in empirical p K a predictions. J.
Chem. Theory Comput..

[26] Reis P. P. S., Vila-Vicosa D., Rocchia W., Machuqueiro M. (2020). PypKa: A flexible
python module for poisson-boltzmann-based p K a calculations. J. Chem. Inf. Model..

[27] Cai Z., Luo F., Wang Y., Li E., Huang Y. (2021). Protein pKa
Prediction
with Machine Learning. ACS Omega.

[28] Cai Z., Liu T., Lin Q., He J., Lei X., Luo F., Huang Y. (2023). Basis for accurate
protein pKa prediction with machine learning. J. Chem. Inf. Model..

[29] Abraham M. J., Murtola T., Schulz R., Páll S., Smith J. C., Hess B., Lindahl E. (2015). GROMACS: High performance
molecular simulations through multi-level parallelism from laptops
to supercomputers. SoftwareX.

[30] Páll, S. ; Abraham, M. J. ; Kutzner, C. ; Hess, B. ; Lindahl, E. Tackling Exascale Software Challenges in Molecular Dynamics Simulations with GROMACS. Solving Software Challenges for Exascale. In Lecture Notes in Computer Science; Springer Nature, 2015; Vol. 8759, pp 3–27 10.1007/978-3-319-15976-8_1.

[31] Jo S., Kim T., Iyer V. G., Im W. (2008). CHARMM-GUI: a web-based graphical
user interface for CHARMM. J. Comput. Chem..

[32] Tian C., Kasavajhala K., Belfon K. A., Raguette L., Huang H., Migues A. N., Bickel J., Wang Y., Pincay J., Wu Q., Simmerling C. (2019). ff19SB: amino-acid-specific protein backbone parameters
trained against quantum mechanics energy surfaces in solution. J. Chem. Theory Comput..

[33] Lee J., Hitzenberger M., Rieger M., Kern N. R., Zacharias M., Im W. (2020). CHARMM-GUI supports the Amber force fields. J. Chem. Phys..

[34] Li P., Song L. F., Merz K. M. (2015). Parameterization
of highly charged metal ions using the 12–6-4 LJ-type nonbonded
model in explicit water. J. Phys. Chem. B.

[35] Migliorati V., Serva A., Terenzio F. M., D’Angelo P. (2017). Development
of Lennard-Jones and Buckingham potentials for lanthanoid ions in
water. Inorg. Chem..

[36] Wang J., Wang W., Kollman P. A., Case D. A. (2006). Automatic atom type
and bond type perception in molecular mechanical calculations. J. Mol. Graphics Model..

[37] Jakalian A., Bush B. L., Jack D. B., Bayly C. I. (2000). Fast, efficient
generation of high-quality atomic charges. AM1-BCC model: I. Method. J. Comput. Chem..

[38] Jakalian A., Jack D. B., Bayly C. I. (2002). Fast, efficient
generation of high-quality
atomic charges. AM1-BCC model: II. Parameterization and validation. J. Comput. Chem..

[39] Bussi G., Donadio D., Parrinello M. (2007). Canonical sampling through velocity
rescaling. J. Chem. Phys..

[40] Bernetti M., Bussi G. (2020). Pressure control using
stochastic cell rescaling. J. Chem. Phys..

[41] Darden T., York D., Pedersen L. (1993). Particle mesh Ewald:
An N log (N) method for Ewald sums in large systems. J. Chem. Phys..

[42] Essmann U., Perera L., Berkowitz M. L., Darden T., Lee H., Pedersen L. G. (1995). A smooth particle mesh Ewald method. J. Chem. Phys..

[43] Hess B., Bekker H., Berendsen H. J., Fraaije J. G. (1997). LINCS: A linear
constraint solver for molecular simulations. J. Comput. Chem..

[44] Hess B. P. (2008). P-LINCS:
A parallel linear constraint solver for molecular simulation. J. Chem. Theory Comput..

[45] Wang L., Hibino A., Suganuma S., Ebihara A., Iwamoto S., Mitsui R., Tani A., Shimada M., Hayakawa T., Nakagawa T. (2020). Preference for particular lanthanide species and thermal
stability of XoxFs in Methylorubrum extorquens strain AM1. Enzyme Microb. Technol..

[46] Schmitz R. A., Picone N., Singer H., Dietl A., Seifert K.-A., Pol A., Jetten M. S. M., Barends T. R. M., Daumann L. J., den Camp H. J. M. O. (2021). Neodymium
as Metal Cofactor for Biological Methanol Oxidation: Structure and
Kinetics of an XoxF1-Type Methanol Dehydrogenase. mBio.

[47] Daura X., Gademann K., Jaun B., Seebach D., Van Gunsteren W. F., Mark A. E. (1999). Peptide folding: when simulation meets experiment. Angew. Chem., Int. Ed..

[48] Mehmood, R. ; Kulik, H. J. Enzyme Engineering: Methods and Protocols; Springer, 2021; pp 227–248.

[49] Neese F. (2025). Software update:
The ORCA program system—version 6.0. WIREs Comput. Mol. Sci..

[50] Neese F. (2023). The SHARK
integral generation and digestion system. J.
Comput. Chem..

[51] Mardirossian N., Head-Gordon M. (2014). *ω*B97X-V: A 10-parameter, range-separated
hybrid, generalized gradient approximation density functional with
nonlocal correlation, designed by a survival-of-the-fittest strategy. Phys. Chem. Chem. Phys..

[52] Weigend F., Ahlrichs R. (2005). Balanced basis sets of split valence, triple zeta valence
and quadruple zeta valence quality for H to Rn: Design and assessment
of accuracy. Phys. Chem. Chem. Phys..

[53] Caldeweyher E., Bannwarth C., Grimme S. (2017). Extension of the D3 dispersion coefficient
model. J. Chem. Phys..

[54] Caldeweyher E., Ehlert S., Hansen A., Neugebauer H., Spicher S., Bannwarth C., Grimme S. (2019). A generally applicable
atomic-charge dependent London dispersion correction. J. Chem. Phys..

[55] Müller M., Hansen A., Grimme S. (2023). *ω*B97X-3c:
A composite range-separated hybrid DFT method with a molecule-optimized
polarized valence double-*ζ* basis set. J. Chem. Phys..

[56] Neese F., Wennmohs F., Hansen A., Becker U. (2009). Efficient, approximate
and parallel Hartree-Fock and hybrid DFT calculations. A ‘chain-of-spheres’
algorithm for the Hartree-Fock exchange. Chem.
Phys..

[57] Helmich-Paris B., de Souza B., Neese F., Izsák R. (2021). An improved
chain of spheres for exchange algorithm. J.
Chem. Phys..

[58] Neese F. (2003). An improvement
of the resolution of the identity approximation for the formation
of the Coulomb matrix. J. Comput. Chem..

[59] Stoychev G. L., Auer A. A., Neese F. (2017). Automatic
generation of auxiliary
basis sets. J. Chem. Theory Comput..

[60] Colinet P., Neese F., Helmich-Paris B. (2025). Improving
the efficiency of electrostatic
embedding using the Fast Multipole Method. J.
Comput. Chem..

[61] Kulik H. J., Luehr N., Ufimtsev I. S., Martinez T. J. (2012). Ab initio quantum
chemistry for protein structures. J. Phys. Chem.
B.

[62] Kulik H. J., Zhang J., Klinman J. P., Martinez T. J. (2016). How large should
the QM region be in QM/MM calculations? The case of catechol O-methyltransferase. J. Phys. Chem. B.

[63] Peng D., Middendorf N., Weigend F., Reiher M. (2013). An efficient
implementation
of two-component relativistic exact-decoupling methods for large molecules. J. Chem. Phys..

[64] Franzke Y. J., Treß R., Pazdera T. M., Weigend F. (2019). Error-consistent segmented
contracted all-electron relativistic basis sets of double-and triple-zeta
quality for NMR shielding constants. Phys. Chem.
Chem. Phys..

[65] Brandt F., Jacob C. R. (2022). Systematic QM region
construction in QM/MM calculations
based on uncertainty quantification. J. Chem.
Theory Comput..

[66] Brandt F., Jacob C. R. (2023). Protein network centralities as descriptor
for QM region
construction in QM/MM simulations of enzymes. Phys. Chem. Chem. Phys..

[67] Brandt F., Jacob C. R. (2023). Efficient automatic construction of atom-economical
QM regions with point-charge variation analysis. Phys. Chem. Chem. Phys..

[68] Marenich A. V., Cramer C. J., Truhlar D. G. (2009). Universal solvation model based on
solute electron density and on a continuum model of the solvent defined
by the bulk dielectric constant and atomic surface tensions. J. Phys. Chem. B.

[69] Case, D. ; Aktulga, H. M. ; Belfon, K. ; Cerutti, D. S. AMBER 2024 2024 http://ambermd.org.

[70] Salomon-Ferrer R., Götz A. W., Poole D., Le Grand S., Walker R. C. (2013). Routine
Microsecond Molecular Dynamics Simulations with AMBER on GPUs. 2.
Explicit Solvent Particle Mesh Ewald. J. Chem.
Theory Comput..

[71] Giese T. J., Zeng J., Lerew L., McCarthy E., Tao Y., Ekesan c., York D. M. (2024). Software
Infrastructure for Next-Generation
QM/MM-ΔMLP Force Fields. J. Phys. Chem.
B.

[72] Bannwarth C., Ehlert S., Grimme S. (2019). GFN2-xTB-An Accurate and Broadly
Parametrized Self-Consistent Tight-Binding Quantum Chemical Method
with Multipole Electrostatics and Density-Dependent Dispersion Contributions. J. Chem. Theory Comput..

[73] Götz A. W., Clark M. A., Walker R. C. (2014). An extensible interface
for QM/MM
molecular dynamics simulations with AMBER. J.
Comput. Chem..

[74] Shiery R.
C., Fulton J. L., Balasubramanian M., Nguyen M.-T., Lu J.-B., Li J., Rousseau R., Glezakou V.-A., Cantu D. C. (2021). Coordination sphere
of lanthanide aqua ions resolved with ab initio molecular dynamics
and X-ray absorption spectroscopy. Inorg. Chem..

[75] Alanís-Manzano E. I., León-Pimentel C., Maron L., Ramírez-Solís A., Saint-Martin H. (2024). Exploring the dynamic coordination sphere of lanthanide
aqua ions: insights from r2SCAN-3c Composite-DFT Born–Oppenheimer
molecular dynamics studies. ACS Omega.

[76] Danaf N. A., Kretzschmar J., Jahn B., Singer H., Pol A., den Camp H. J. O., Steudtner R., Lamb D. C., Drobot B., Daumann L. J. (2022). Studies
of pyrroloquinoline quinone species in solution
and in lanthanide-dependent methanol dehydrogenases. Phys. Chem. Chem. Phys..

[77] Driscoll D.
M., White F. D., Pramanik S., Einkauf J. D., Ravel B., Bykov D., Roy S., Mayes R. T., Delmau L. H., Cary S. K. (2024). Observation
of a promethium complex in solution. Nature.

[78] Peters J. A., Djanashvili K., Geraldes C. F., Platas-Iglesias C. (2020). The chemical
consequences of the gradual decrease of the ionic radius along the
Ln-series. Coord. Chem. Rev..

[79] Grimmel S., Schoendorff G., Wilson A. K. (2016). Gauging the performance of density
functionals for lanthanide-containing molecules. J. Chem. Theory Comput..

[80] Carrillo-Tripp M., Saint-Martin H., Ortega-Blake I. (2004). Minimalist
Molecular Model for Nanopore
Selectivity. Phys. Rev. Lett..

[81] Carrillo-Tripp M., San-Román M. L., Hernańdez-Cobos J., Saint-Martin H., Ortega-Blake I. (2006). Ion hydration in nanopores and the molecular basis
of selectivity. Biophys. Chem..

[82] Friedman R. (2021). Preferential
binding of lanthanides to methanol dehydrogenase evaluated with density
functional theory. J. Phys. Chem. B.

