# Supplementary material for: QM/MM Reveals Coordination Shift behind Early Lanthanide Preference in Methanol Dehydrogenase XoxF
Source: J Phys Chem B. 2026 Aug 27;130(36):9070–83. doi: 10.1021/acs.jpcb.6c03103 (PMC13573614; doi:10.1021/acs.jpcb.6c03103)
Supplement: Supplementary file 1 [file jp6c03103_si_001.pdf]

Supplementary Information: QM/MM Reveals Coordination Shift Behind Early Lanthanide Preference in Methanol Dehydrogenase XoxF

Emiliano Isaías Alanís-Manzano,*,† José Luis Velázquez-Libera,‡ César Millan Pacheco,¶ Alejandro Ramírez-Solís,§ and Humberto Saint-Martin†

†*Instituto de Ciencias Físicas, Universidad Nacional Autónoma de México, Cuernavaca Morelos 62210 México.*

‡*Departamento de Bioinformática, Centro de Bioinformática, Simulación y Modelado (CBSM), Facultad de Ingeniería, Universidad de Talca, Talca 3460000, Chile*

¶*Facultad de Farmacia, Universidad Autónoma del Estado de Morelos, Cuernavaca, Morelos 62209 México*

§*Centro de Investigación en Ciencias, Universidad Autónoma del Estado de Morelos, Cuernavaca, Morelos 62209 México*

E-mail: emiliano@icf.unam.mx

S1. Ionization state of the Enzyme

Protonation states of titratable residues were initially predicted using the H++ web server. While these predictions are trustworthy for residues distant from the metal center, they should be treated with caution near the active site because H++ does not account for the Ln^{3+} ion or the PQQ cofactor.

For residues not directly coordinated to PQQ or the lanthanide, the protonation states

were cross-validated using PropKa3, DelphiPka, and DeepKa. All methods yielded consistent results.

The acidic amino acid residues that coordinate the lanthanide are treated as deprotonated. Furthermore, analysis of the PQQ cofactor's first neighbors shows that the carboxylate group of Glu55 and the PQQ's O2 atom are both solvent-inaccessible and positioned at a hydrogen-bonding distance necessitating that one of them is protonated to stabilize their interaction. Similarly, the carboxylate of Asp388 is located to form a hydrogen bond with the PQQ's C4 carbonyl oxygen within a solvent-shielded environment, suggesting Asp388 is also protonated. This structural assessment is further supported by exploratory MD simulations, wherein exploratory models with either ASP388 or GLU55 in a deprotonated state resulted in large-amplitude residue movements and a marked destabilization of the PQQ cofactor within the active site. Consequently, we consider that both ASP388 and GLU55 are protonated, while all other amino acid residues maintain their standard protonation states at pH 7.4.

S2. Classical Molecular Dynamics details

It was necessary to use harmonic restraints to keep the substrate in the active site. Two restraints were used, one for each distance: Ce–OMeOH and Ce–OD2Asp299 (where $OD2_{Asp299}$ is the oxygen of the carboxyl group of Asp299 that is not coordinated to Ce^{3+} in the crystal structure). A force constant of 2000 $kJ\,mol^{-1}\,nm^{-2}$ was used for the restraints during the equilibration and production phases of the molecular dynamics simulation. This approach has already been utilized to sample reactive conformations that might be short lived[1]

The RMSDs are extremely low, *ca.* 0.8 Å, for the three replica. No positional restaints were imposed on the protein back-bone. This might be an artifact of the FF19SB forcefield. In an exploratory MD simulation, we extended simulation time to 500 ns and no significant change was observed in the CA-RMSD. Nevertheless, as discussed in the main text our goal is to simply obtain a enzyme-substrate complex consistent with the physiological conditions

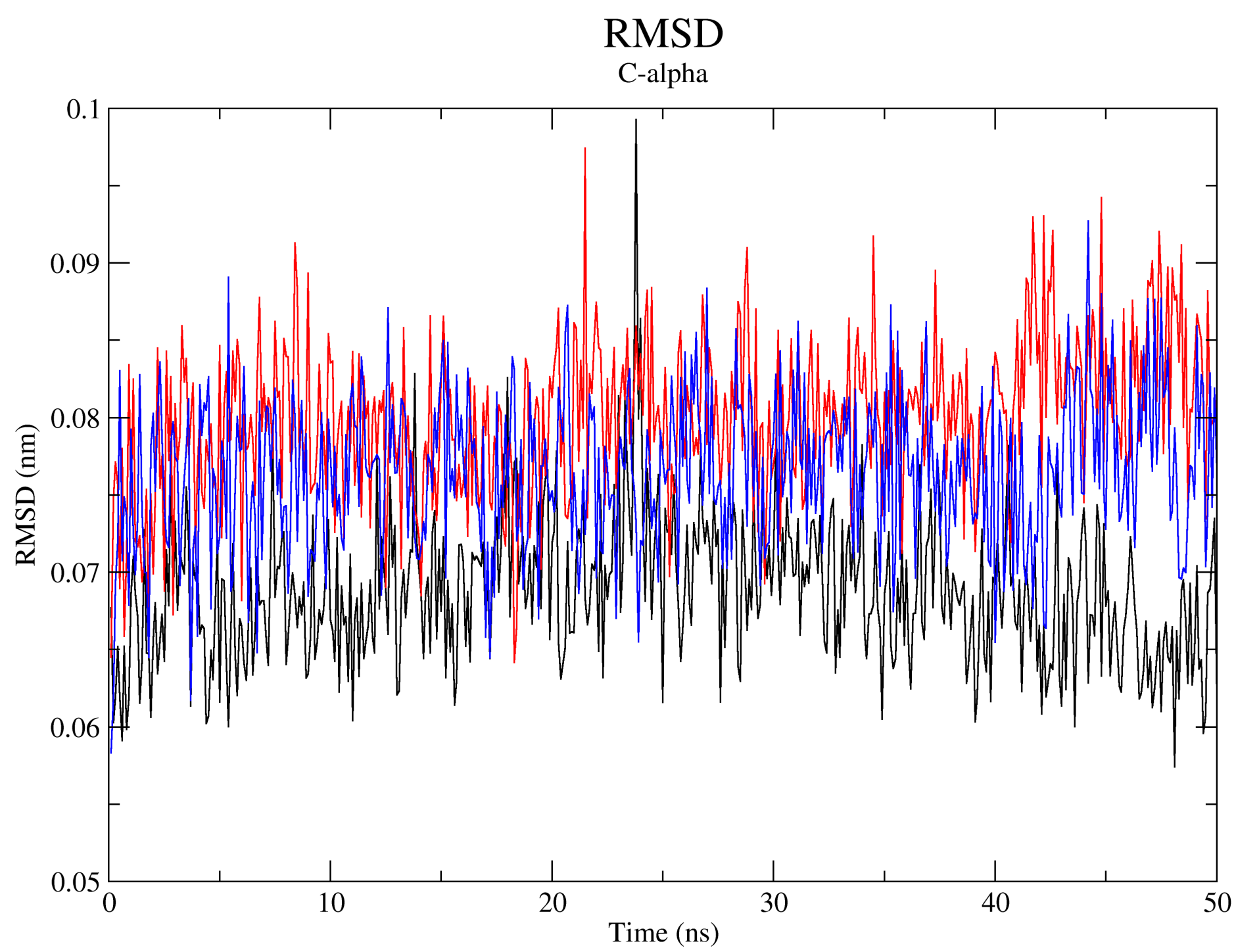

Figure S1: RMSD of the protein backbone of the three 50 ns replicas of the MD simulations of the Enzyme-Substrate complex

for further QM/MM geometry optimizations.

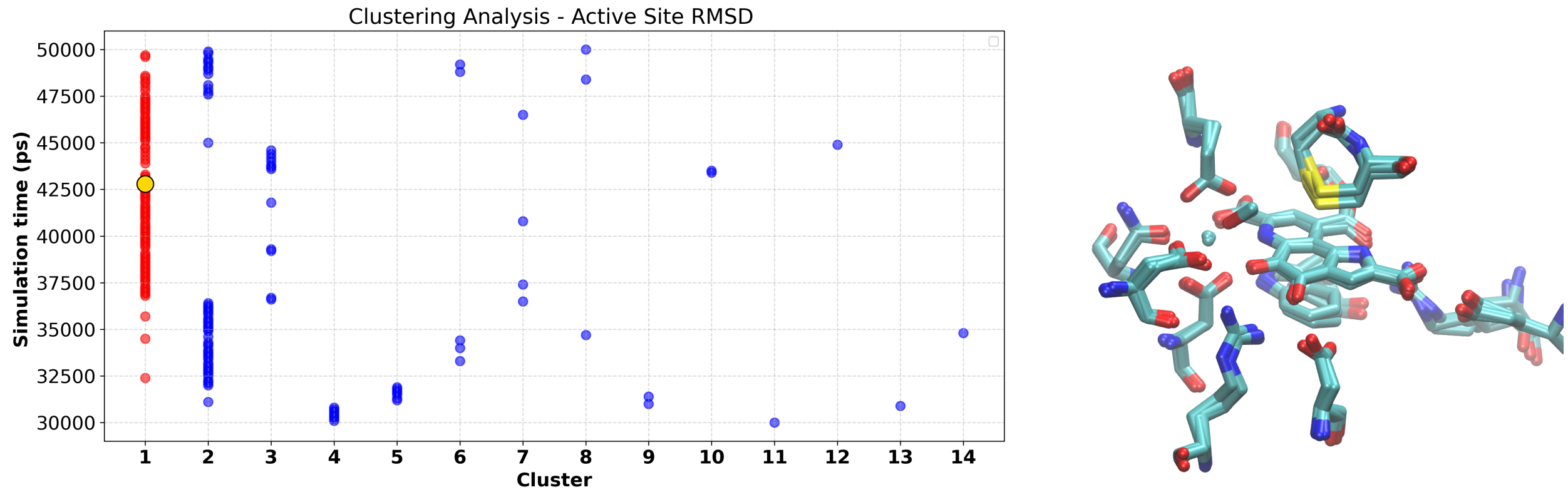

Figure S2: Left panel: Clustering analysis results over the last 20 ns of the productive MD simulation. All frames were derived in 14 clusters and they are represented to the most to the least populated ones. The representative structure used in this study is represented by the yellow point. Right Panel: Superoposition of the centroid structures of the first three clusters.

To obtain a representative structure from the 50 ns classical MD trajectory, a clustering analysis of the frames was performed using the RMSD of the heavy atoms from the residues near the metal and the PQQ cofactor, these residues are shown in (FIG S2. The selected representative structure (FIG S2) was utilized for the QM/MM system preparation.

S3. QM/MM interface

The QM region comprised the side-chain functional groups of residues ASP301, ASP299, ASH388, SER169, CYX104, CYX105, GLU172, GLH55, ARG326, ARG110, and TRP238. Link atoms were used to cap the valencies at the boundary to the MM region, with cuts made at the following positions to retain the side-chain moieties in the QM layer:

- **Cα–Cβ bond:** ASP301, ASP299, ASH388, SER169, CYX104, CYX105
- **Cγ–Cδ bond:** GLU172, GLH55, ARG326, ARG110
- **Cβ–Cγ bond:** TRP238

For single-point calculations with larger QM regions, the placement of link atoms was adjusted to improve efficiency. Link atoms were placed on the Cα-C and Cα-N bonds only including the α-carbon atom in the QM region for single QM amino acids, while also including the remaining backbone atoms between two subsequent QM amino acids to reduce the number of link atoms.

S4. QM region justification

The selection of the QM region is a crucial step in the workflow of computational enzymology. Including residues only by considering their distances to the active site is a naive approach that is almost obsolete nowadays. Therefore, systematic methods have been developed to exclusively detect crucial residues. Typically, the systematic methods are based on the electrostatic effect of single aminoacids on the active site.

Here we utilize a recent approach of Brandt and Jacob, the Point Charge Variation Analysis (PCVA).[2,3] Which requires only a geometry optimization of the target system with a minimal QM region including substrates, which is followed by single-point calculations for the point-charge variation of each amino acid. This gives a systematic framework to discern important electrostatic residues

This approach is combined with Protein network centralities,[4] also reported by Brandt and Jacob, in order to identify important residues that are missed in purely electrostatic approaches.

Following Brandt and Jacob we use the b-centrality descriptor to rank non-electrostatic important residues.

The saa-PCVA was implemented in python. We adapted this method by including seven structurally resolved water molecules in our charge variation analysis, granting them the same consideration as amino acid residues. These waters are all positioned close to the active site. A chain of five waters mediates interactions with GLU172, the disulfide bridge, and the PQQ's C2-carboxylate group. The two other waters have distinct roles: one bridges

ARG326 and ASH388, and the other hydrogen-bonds specifically with SER169.

The saa-PVCA indicator was further modified by changing the way the distance was calculated in Equation (16) of Ref.[2] Instead of using the center-of-mass distance from the cofactors (Ce3+/PQQ) to a residue (as in the original method) we now employ the minimum inter-atomic distance. This calculates the shortest distance between any atom belonging to the cofactors and any atom of the residue or water in question.

The minimal QM region considered for the saa-PCVA was the Ce^3+, PQQ, MeOH and the first shell (FS) residues ASP299, GLU172, ASN256 and ASP301.

Four of the seven water molecules rank as highly as the top 16 amino acid residues within the modified saa-PCVA. Nonetheless, we included all water molecules in the QM region because the remaining three, while not high-ranked, appear to play important roles: one participates in a salt bridge with GLU172, another interacts with the C7-carboxylate of PQQ, and the last one bridges ARG326 and ASP388.

Now for the protein network analysis, the workflow described by Brandt and Jacob was followed.[4] In short, we utilized our 50 ns MD trajectory as a basis for graph construction. The WISP script was utilized for correlation matrix and contact map construction. Then tne networkx method was utilized for calculating b-centralities.

The final QM region includes the six highest-ranked residues from the PCVA analysis, supplemented by TRP238, TRP260, TRP467, and TRP532, which were also highly ranked by the b-centrality descriptor. Residues TRP240 and TYR252, identified by b-centrality, were excluded due to their distance (greater than 9 Å) from the active site. Though these residues might be an indication of long-range allosteric interactions, for our current study of the coordination properties of the active site this residues will be ommited. Although not detected by any automated method, the disulfide bridge CYX104–CYX105 and residue ASH388 were manually added to the QM region. ASH388 was included for its steric importance, as it forms a hydrogen bond with the O4-carbonyl oxygen of PQQ. The seven crystallographic water molecules discussed previously were also incorporated.

Overall the $\boldsymbol{R}_{\text{En}}$ QM region consists of 17 residues and 7 water molecules.

As a sanity check, given that the RMSD plot (FIG: S1) shows a very rigid protein. We carried out a 1.2 μs MD simulation with the ff14SB forcefield for the protein atoms and TIP3P water model, the other forcefield parameters of cofactors, substrate, and Ln^{3+} atoms remained the same. Within this Force Field the protein structure is still quite rigid, with a mean RMSD-CA of approximately 1.3 Å. We repeated the protein network analysis with this larger trajectory and the results are essentially unchanged. This is consistent with studies on lanthanide-coordinated enzymes that show that lighter lanthanides confer high catalytic activity and thermal stability to the active sites of these pyrroloquinoline quinone-dependent proteins, allowing them to remain stable and active under elevated temperatures –from 50°C to 60°C– that denature alternative calcium-dependent systems.[5,6]

Table S1: Aminoacid residues included in the QM region utilized for the energetic analysis.

PCVA	b-centralities	FS + extra residues[a]
ARG110	TRP532	GLU172
GLH55	TRP467	ASN256
THR154	TRP238	ASP299
SER169	TRP260	ASP301
ARG326	-	CYX104
GLY170	-	CYX105
-	-	ASH388

[a] First shell (FS) residues as well as the disulfide bridge CYX104-CYX105 and ASH388.

The aminoacid residues and water molecules included in $\boldsymbol{R}_{\text{opt}}$ and $\boldsymbol{R}_{\text{En}}$ are:

- $\boldsymbol{R}_{\text{opt}}$: GLH55, CYX104, CYX105, ARG110, SER169, TRP288, ARG326, ASH388, ASN256, ASP299, ASP301, GLU172 + WAT642 + WAT661 + WAT639 + WAT662

- $\boldsymbol{R}_{\text{En}}$: $\boldsymbol{R}_{\text{opt}}$ + TRP532 + TRP467 + TRP260 + THR154 + GLY170 + WAT632 + WAT721 + WAT603

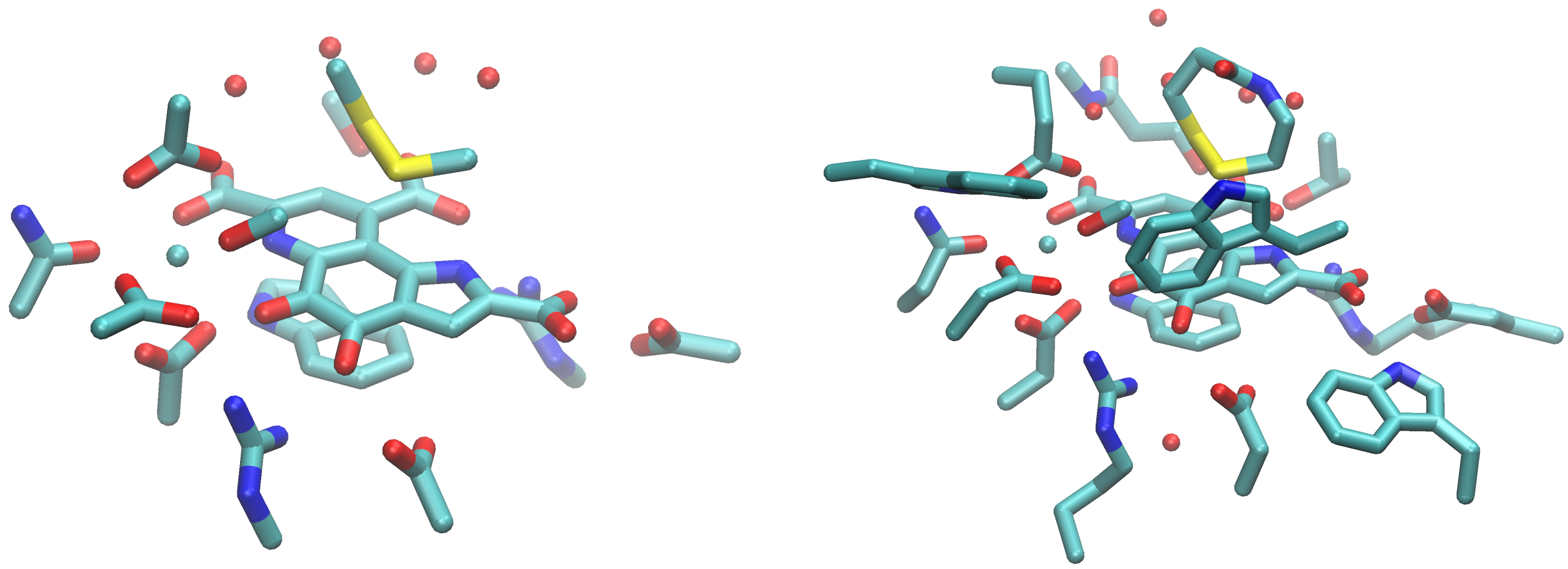

Figure S3: Visual representation of $\boldsymbol{R}_{\mathrm{opt}}$ and $\boldsymbol{R}_{\mathrm{En}}$ for the Enzyme-Substrate complex.

S5. Optimal DFT distances

- Average coordination shell distance $\langle r \rangle$: For enzyme-substrate bound complexes the average is obtained considering ASP301 distance up tp Gd, from Tb to Lu this distance is excluded, the O-MeOH distance is always included in the average. For the substrate free complexes all 10 distances are always considered as no coordination number change is observed.

- The contraction of the coordination shell ΔCS is the reduction in the average first coordination shell Ln–ligand distance relative to La. Δ CS = $\langle r \rangle_{\mathrm{Ln}}$ - $\langle r \rangle_{\mathrm{La}}$, where Ln = Ce - Lu.

Table S2: First-shell coordination distances (Å) to the Ln^{3+} ion (La-Gd). ωB97X-D4rev/def2-SVP:FF19SB optimized structures of the Ln^{3+}-MDH enzyme-substrate complexes

Enzyme-Substrate complex - La to Gd								
	La	Ce	Pr	Nd	Pm	Sm	Eu	Gd
172GluOE1	2.62	2.59	2.59	2.57	2.56	2.55	2.53	2.53
172GluOE2	2.66	2.63	2.63	2.63	2.61	2.60	2.60	2.59
256AsnOD1	2.49	2.47	2.46	2.44	2.43	2.41	2.39	2.38
299AspOD1	2.52	2.50	2.49	2.48	2.47	2.46	2.45	2.44
299AspOD2	3.68	3.63	3.67	3.67	3.66	3.66	3.65	3.64
301AspOD1	2.71	2.69	2.69	2.67	2.68	2.68	2.68	2.69
301AspOD2	2.66	2.62	2.62	2.61	2.59	2.57	2.55	2.53
PQQO7	2.58	2.56	2.55	2.53	2.52	2.51	2.50	2.48
PQQN6	2.85	2.81	2.83	2.82	2.81	2.79	2.78	2.77
PQQO5	2.83	2.80	2.83	2.84	2.83	2.83	2.83	2.83
O(MeOH)	2.58	2.55	2.54	2.53	2.51	2.49	2.48	2.47
$\langle r \rangle$	2.65	2.62	2.62	2.61	2.60	2.59	2.58	2.57
ΔCS	0	-0.03	-0.03	-0.04	-0.05	-0.06	-0.07	-0.08
NC =	10	10	10	10	10	10	10	10

Table S3: First-shell coordination distances (Å) to the Ln^{3+} ion (Tb-Lu). ωB97X-D4rev/def2-SVP:FF19SB optimized structures of the Ln^{3+}-MDH enzyme-substrate complexes.

Enzyme-Substrate complex - Tb to Lu							
	Tb	Dy	Ho	Er	Tm	Yb	Lu
172GluOE1	2.48	2.47	2.46	2.44	2.43	2.42	2.42
172GluOE2	2.53	2.52	2.51	2.50	2.49	2.48	2.48
256AsnOD1	2.39	2.38	2.36	2.35	2.34	2.33	2.32
299AspOD1	2.41	2.40	2.39	2.38	2.37	2.37	2.36
299AspOD2	3.64	3.64	3.64	3.63	3.63	3.62	3.62
301AspOD1	3.55	3.56	3.57	3.57	3.58	3.58	3.58
301AspOD2	2.31	2.29	2.28	2.26	2.25	2.23	2.23
PQQO7	2.44	2.43	2.41	2.40	2.39	2.37	2.36
PQQN6	2.77	2.77	2.77	2.77	2.78	2.78	2.78
PQQO5	2.92	2.95	2.98	3.00	3.03	3.06	3.08
O(MeOH)	2.42	2.40	2.38	2.37	2.35	2.33	2.32
$\langle r \rangle$	2.52	2.51	2.50	2.50	2.49	2.49	2.48
ΔCS	-0.13	-0.14	-0.15	-0.15	-0.16	-0.16	-0.17
NC =	9	9	9	9	9	9	9

Table S4: First-shell coordination distances (Å) to the Ln^{3+} ion (La-Gd). ωB97X-D4rev/def2-SVP:FF19SB optimized structures of the Ln^{3+}-MDH enzyme without MeOH.

Enzyme without Substrate - La to Gd								
	La	Ce	Pr	Nd	Pm	Sm	Eu	Gd
172GluOE1	2.57	2.55	2.54	2.52	2.51	2.49	2.48	2.47
172GluOE2	2.67	2.66	2.65	2.64	2.63	2.62	2.60	2.59
256AsnOD1	2.49	2.48	2.46	2.44	2.43	2.42	2.40	2.39
299AspOD1	2.61	2.60	2.59	2.58	2.57	2.56	2.55	2.54
299AspOD2	2.59	2.58	2.57	2.55	2.53	2.52	2.50	2.49
301AspOD1	2.66	2.64	2.63	2.61	2.59	2.58	2.56	2.54
301AspOD2	2.69	2.69	2.68	2.67	2.66	2.65	2.65	2.64
PQQO7	2.60	2.59	2.57	2.56	2.54	2.53	2.51	2.50
PQQN6	2.98	2.99	2.99	2.99	2.99	2.99	3.00	3.00
PQQO5	2.89	2.89	2.90	2.91	2.93	2.94	2.96	2.98
$\langle r \rangle$	2.68	2.67	2.66	2.65	2.64	2.63	2.62	2.61
ΔCS	0	-0.01	-0.02	-0.02	-0.04	-0.05	-0.06	-0.07
NC =	10	10	10	10	10	10	10	10

Table S5: First-shell coordination distances (Å) to the Ln^{3+} ion (Tb-Lu). ωB97X-D4rev/def2-SVP:FF19SB optimized structures of the Ln^{3+}-MDH enzyme without substrate.

Enzyme without Substrate - Tb to Lu							
	Tb	Dy	Ho	Er	Tm	Yb	Lu
172GluOE1	2.45	2.44	2.42	2.41	2.40	2.39	2.38
172GluOE2	2.58	2.57	2.56	2.55	2.55	2.54	2.53
256AsnOD1	2.38	2.37	2.36	2.34	2.33	2.31	2.30
299AspOD1	2.53	2.53	2.52	2.51	2.51	2.49	2.49
299AspOD2	2.48	2.46	2.44	2.43	2.42	2.41	2.40
301AspOD1	2.53	2.50	2.49	2.47	2.46	2.44	2.43
301AspOD2	2.63	2.62	2.62	2.61	2.61	2.62	2.63
PQQO7	2.48	2.47	2.45	2.43	2.43	2.40	2.39
PQQN6	3.01	3.02	3.03	3.04	3.05	3.07	3.08
PQQO5	3.00	3.03	3.06	3.09	3.10	3.16	3.18
$\langle r \rangle$	2.61	2.60	2.60	2.59	2.59	2.58	2.58
ΔCS	-0.07	-0.08	-0.08	-0.09	-0.09	-0.10	-0.10
NC =	10	10	10	10	10	10	10

S6. Coordination-mode populations from the DFT/MM MD simulations

This section collects the numerical values represented in Figure 5 of the main text. The populations were obtained from the 2 ps DFT/MM MD trajectories computed at the LC-ECP/ωB97X-D4rev/def2-SVP/FF14SB level of theory at 320 K, one trajectory per Ln^{3+} cation, after discarding the first 100 frames of each trajectory as equilibration.

A donor atom was assigned to the first coordination shell when its distance to the metal center was equal to or shorter than 3.1 Å. For the carboxylate residues Glu172, Asp299 and Asp301, the bidentate mode additionally required the difference between the two Ln–O distances to be equal to or smaller than 0.5 Å; carboxylates satisfying the distance cutoff for only one oxygen, or presenting a difference larger than 0.5 Å between the two Ln–O distances, were counted as monodentate. The denticity of the PQQ cofactor was defined as the number of its O5, N6 and O7A atoms within the cutoff. Asn256 and the methanol substrate contribute at most one donor atom each. The total coordination number is the sum of these six contributions, so that its maximum attainable value is eleven. Water molecules were not found within the first coordination shell of any cation during these simulations.

Table S6: Populations (%) of the coordination modes of the carboxylate residues Glu172, Asp299 and Asp301 and of the PQQ cofactor, obtained from the 2 ps DFT/MM MD simulations of the Ln^{3+}–MDH-ES complexes at 320 K. Mono, bi and tri denote monodentate, bidentate and tridentate coordination, respectively. Rows may not add up to 100% because configurations in which a ligand left the first coordination shell are not listed.

	Glu172		Asp299		Asp301		PQQ	
Ln	mono	bi	mono	bi	mono	bi	bi	tri
La	6	94	0	100	11	89	17	74
Ce	20	80	38	62	42	58	3	97
Pr	44	56	24	76	43	57	17	81
Nd	14	86	76	24	23	77	11	84
Pm	6	94	94	6	42	58	10	88
Sm	92	8	14	86	29	71	15	70
Eu	6	94	79	21	35	65	22	76
Gd	30	70	82	18	60	40	14	77
Tb	0	100	95	5	75	25	14	86
Dy	45	55	51	49	86	14	14	81
Ho	51	49	89	11	59	41	13	85
Er	49	51	58	42	66	34	33	59
Tm	6	94	89	11	96	4	4	94
Yb	32	68	93	7	95	5	25	67
Lu	43	57	100	0	79	21	33	67

Table S7: Populations (%) of the total coordination number (CN) of the Ln^{3+} cation, obtained from the 2 ps DFT/MM MD simulations of the Ln^{3+}–MDH-ES complexes at 320 K. The coordination number counts the donor atoms of Asn256, Glu172, Asp299, Asp301, the PQQ cofactor and the methanol substrate, so that its maximum attainable value is eleven. The last two columns give the combined populations of the high- and low-coordination regimes.

Ln	CN=7	CN=8	CN=9	CN=10	CN=11	CN≥10	CN≤8
La	0	2	9	28	61	89	2
Ce	0	3	22	52	24	76	3
Pr	0	9	31	43	17	60	9
Nd	2	2	28	61	6	67	4
Pm	1	7	41	48	3	51	8
Sm	0	15	50	36	0	36	15
Eu	0	4	48	40	8	48	4
Gd	0	29	48	24	0	24	29
Tb	0	14	56	30	0	30	14
Dy	3	16	66	16	0	16	19
Ho	1	29	54	14	1	15	30
Er	2	34	47	17	0	17	36
Tm	0	8	84	7	1	8	8
Yb	3	57	40	1	0	1	60
Lu	6	44	47	2	0	2	50

S7. Relative MDH affinities

Table S8: Computed affinity values (kcal/mol) of MDH for its substrate and for lanthanide cofactor (Ln) realtive to Ln = La. Comparison between all-electron (X2C) and effective core potential (LCECP) methods. For comparison Y^{3+} ECP data is also shown.

	MeOH		Ln^{3+}	
Ln^{3+}	X2C	LCECP	X2C	LCECP
Ce	−3.15	−2.72	−0.03	0.58
Pr	−0.03	−0.08	0.30	1.69
Nd	1.38	1.20	2.19	2.91
Pm	−0.16	−0.38	2.15	3.53
Sm	0.53	−0.25	2.51	4.94
Eu	−4.93	−4.35	4.66	5.46
Gd	−4.60	−4.37	6.71	5.97
Tb	0.87	2.12	8.39	7.81
Dy	20.90	20.96	−12.70	−11.78
Ho	18.87	20.21	−9.09	−10.43
Er	19.59	20.10	−9.90	−10.37
Tm	17.72	19.18	−8.61	−9.49
Yb	−1.87	−1.00	10.46	9.66
Lu	−2.32	−1.09	12.79	11.30
Y	–	15.85	–	−10.28

S8. Y^{3+}-MDH Results

To strengthen the conclusion on the relevance of the coordination properties of cations in the enzyme's preference for the lighter Lns, due almost exclusively to their larger size and high coordination numbers, further calculations were performed with Y^{3+}; this rare-earth element belongs to the same chemical group as La^{3+}, but it is lighter and smaller, and its coordination properties are similar to the heavier Lns.

Geometry optimizations of MeOH-bound and MeOH-free Y^{3+}-MDH were carried out using the same computational approach as described in the main text. For the MD phase the non-bonded model with the Lennard-Jones (LJ) parameters from Li *et al.*[7] was employed. With respect to the QM/MM computations For this Y^{3+} ion the Stuttgart-Dresden (SDD)

ECP was employed along its associated def2-SVP basis set.

Something analogous happens with the energetic analysis, the SDD ECP was maintained but the metal center basis set was set to def2-TZVPP. The basis set specifications for the remaining protein atoms and XC functional are the same as explained in the main text.

Table S9: First-shell coordination distances (Å) to the Y^{3+} ion. SDD/ωB97X-D4rev/def2-SVP:FF19SB optimized structures of the Y^{3+}-MDH enzyme with and without substrate.

Y^{3+}- Binding distances (Å)		
	MeOH-bound	MeOH-free
172GluOE1	2.44	2.40
172GluOE2	2.49	2.58
256AsnOD1	2.35	2.34
299AspOD1	2.38	2.51
299AspOD2	3.64	2.43
301AspOD1	3.59	2.47
301AspOD2	2.26	2.62
PQQO7	2.39	2.43
PQQN2	2.77	3.03
PQQO5	3.00	3.07
O(MeOH)	2.37	-

Table S10: Computed affinity values (kcal/mol) of MDH for its substrate and for the rare-earth ion Y^{3+} realtive to La.

Y^{3+} affinities relative to La^{3+}	
$\Delta\mathcal{E}_{\text{MeOH}}$	15.85
$\Delta\mathcal{E}_{Y^{3+}}$	-10.28

References

(1) Santos-Martins, D.; Calixto, A. R.; Fernandes, P. A.; Ramos, M. J. A buried water molecule influences reactivity in α-Amylase on a subnanosecond time scale. *ACS Catalysis* **2018**, *8*, 4055–4063.

(2) Brandt, F.; Jacob, C. R. Systematic QM region construction in QM/MM calculations based on uncertainty quantification. *Journal of Chemical Theory and Computation* **2022**, *18*, 2584–2596.

(3) Brandt, F.; Jacob, C. R. Efficient automatic construction of atom-economical QM regions with point-charge variation analysis. *Physical Chemistry Chemical Physics* **2023**, *25*, 14484–14495.

(4) Brandt, F.; Jacob, C. R. Protein network centralities as descriptor for QM region construction in QM/MM simulations of enzymes. *Physical Chemistry Chemical Physics* **2023**, *25*, 20183–20188.

(5) Wang, L.; Hibino, A.; Suganuma, S.; Ebihara, A.; Iwamoto, S.; Mitsui, R.; Tani, A.; Shimada, M.; Hayakawa, T.; Nakagawa, T. Preference for particular lanthanide species and thermal stability of XoxFs in Methylorubrum extorquens strain AM1. *Enzyme and Microbial Technology* **2020**, *136*, 109518.

(6) Schmitz, R. A.; Picone, N.; Singer, H.; Dietl, A.; Seifert, K.-A.; Pol, A.; Jetten, M. S. M.; Barends, T. R. M.; Daumann, L. J.; Op den Camp, H. J. M. Neodymium as Metal Cofactor for Biological Methanol Oxidation: Structure and Kinetics of an XoxF1-Type Methanol Dehydrogenase. *mBio* **2021**, *12*.

(7) Li, P.; Song, L. F.; Merz Jr, K. M. Parameterization of highly charged metal ions using the 12-6-4 LJ-type nonbonded model in explicit water. *The Journal of Physical Chemistry B* **2015**, *119*, 883–895.
